# Marker-trait association analysis of frost tolerance of 672 worldwide pea (*Pisum sativum* L.) collections

**DOI:** 10.1038/s41598-017-06222-y

**Published:** 2017-07-19

**Authors:** Rong Liu, Li Fang, Tao Yang, Xiaoyan Zhang, Jinguo Hu, Hongyan Zhang, Wenliang Han, Zeke Hua, Junjie Hao, Xuxiao Zong

**Affiliations:** 1 Center for Crop Germplasm Resources/Institute of Crop Sciences, Chinese Academy of Agricultural Sciences, Beijing, 100081 China; 2 Qingdao Academy of Agricultural Sciences, Qingdao, 266100 Shandong China; 3 USDA, Agricultural Research Service, Western Regional Plant Introduction Station, Washington State University, Pullman, WA 99164 USA; 4 Binzhou Academy of Agricultural Sciences, Binzhou, 256600 Shandong China; 5 Laiyang Agricultural Extension Center, Laiyang, 265200 Shandong China

## Abstract

Frost stress is one of the major abiotic stresses causing seedling death and yield reduction in winter pea. To improve the frost tolerance of pea, field evaluation of frost tolerance was conducted on 672 diverse pea accessions at three locations in Northern China in three growing seasons from 2013 to 2016 and marker-trait association analysis of frost tolerance were performed with 267 informative SSR markers in this study. Sixteen accessions were identified as the most winter-hardy for their ability to survive in all nine field experiments with a mean survival rate of 0.57, ranging from 0.41 to 0.75. Population structure analysis revealed a structured population of two sub-populations plus some admixtures in the 672 accessions. Association analysis detected seven markers that repeatedly had associations with frost tolerance in at least two different environments with two different statistical models. One of the markers is the functional marker EST1109 on LG VI which was predicted to co-localize with a gene involved in the metabolism of glycoproteins in response to chilling stress and may provide a novel mechanism of frost tolerance in pea. These winter-hardy germplasms and frost tolerance associated markers will play a vital role in marker-assisted breeding for winter-hardy pea cultivar.

## Introduction

Since Mendel’s laws of genetic heritance, pea (*Pisum sativum* L.) has been one model plant for genetic study^1^. In addition, pea is one of the most economically important legume crops. It can be used both as a grain and a vegetable and provides a high quality source of protein for humans and livestock^2, 3^. Moreover, pea is also considered an environmentally friendly crop due to its nitrogen fixation capacity which can reduce the need for nitrogen fertilizer and greenhouse gas emissions^4, 5^. Pea is classified by end use into dry pea (also known as field pea) and green pea. In 2014, pea ranked the third (following common bean and chickpeas) in dry grain and the second (next to common bean) in green pea in the global production among legume crops, respectively^6^. With the growing market demand, China became the largest green pea and the second largest dry pea producer in the world^6^.

Freezing temperatures influence the growth and reproduction of plants and limit the distribution range of them^7, 8^. During the long-term process of evolution, plants evolved an adaptation to withstand cold which is known as “cold acclimation” induced by low, non-freezing temperatures^9, 10^. However, frost damage will occur resulting in irreversible injury such as destruction of cell membrane system or loss of photosynthetically active tissue when plants are not acclimated^11, 12^. For legumes species, frost stress is also one of the principal limiting abiotic factors affecting their production by damaging seedlings and limiting plant growth^13, 14^. Therefore, striking out in depth research on plant frost tolerance is significant not only in exploring the molecular basis of cold acclimation, but also to practical production of most crops^11^.

Field pea consists of three phenological types: spring, Mediterranean and winter^15^. Currently, autumn sowing for winter types is widely accepted as a way to improve yield potential and stability in pea due to advantages of a higher biomass production through a longer life cycle as well as the avoidance of the drought and the heat stress of late spring^14–16^. Although both spring and winter types of field pea are grown in China, most production of pea is in the semi-tropical climate zone as an upland winter crop^17^. However, severe frost damage in winter or early spring that occurs at the seedling stage may cause high rate of seedling death and much production decrease^18, 19^. Thus, studies on frost tolerance and breeding for winter-hardy cultivars play fundamental roles in the stable increase of pea production^20–23^.

To overcome the frost damage to pea production in winter, some literature on winter hardiness in pea have been struck in previous studies. Field pea was found to exhibit a moderate freezing tolerance with a LT_50_ (temperature that kills 50% of seedlings) of −4.5 °C comparing with some forage legumes^18^, while some winter hardy cultivars of pea were found to be able to adapt to a temperature range between −8 to −12 °C^20, 23^. Moreover, breeding efforts were given to the development of some winter hardy pea cultivars that have already been utilized in the temperate regions of Europe and the USA^21, 24–26^. Recently, winter hardiness was evaluated for a limited 58 accessions of pea germplasm under both field and laboratory conditions in Turkey, and identified genotypes with differential survival at −8 °C among which the higher level of winter hardiness were selected for future cultivar development^20^. Meanwhile, a large-scale evaluation of 3,672 pea germplasm for cold tolerance was conducted in the field in China and found that genotypes from winter production regions showed a higher level of cold tolerance than those from spring production regions and identified a collection of genetic resources for cold-tolerance of pea in China^22^. However, due to the significant effect of environmental heterogeneity in field screening for frost tolerance^15^, multi-location and multi-year field trails are necessary to test the cold tolerance adaptation to a more wide range of these genetic resources in China.

Association mapping, utilizing a broader gene pool including natural population, germplasm collection and breeding cultivars, has emerged as an alternative approach to traditional QTL mapping and provides a powerful tool for identification of novel functional variation and quantitative traits in plants^27, 28^. There are several advantages of association mapping over traditional QTL mapping: (1) utilizing a broader genetic variations for marker-trait correlations, (2) obtaining a higher resolution mapping due to more recombination events in germplasm development history, (3) availability of exploiting previous phenotyping data, and (4) no need to develop costly, labor-intensive and time-consuming biparental populations^28, 29^. Association mapping is generally applied to identify markers useful in marker-assisted selection (MAS) for superior individuals in crop breeding or in cloning functional genes governing important agronomic traits, and has been successfully performed in many crops including maize^30^, soybean^31^, *Aegilops tauschii* ^32^ and also in pea emphasizing on seed nutrients, agronomic and quality traits with germplasm collections^33–35^. However, the marker-trait association of frost tolerance in pea has not been reported yet.

In this study, field screening for winter-hardy germplasms and marker-trait association analysis of frost tolerance in pea were conducted to 672 diverse pea accessions including germplasm collections from China, USA and other countries and a set of previously identified cold tolerant germplasms^22^. Field evaluation data were collected across three locations in Northern China and three growing seasons from 2013 to 2016 and genotype data were based on 267 polymorphic SSR markers. The objectives of this study were as follows: (1) to screen for winter-hardy germplasms in pea, (2) to assess the pattern of genetic variation and population structure, and (3) to identify frost tolerance associated SSR markers to use in MAS pea breeding programs.

## Results

### Phenotype variability

**Figure 1:**
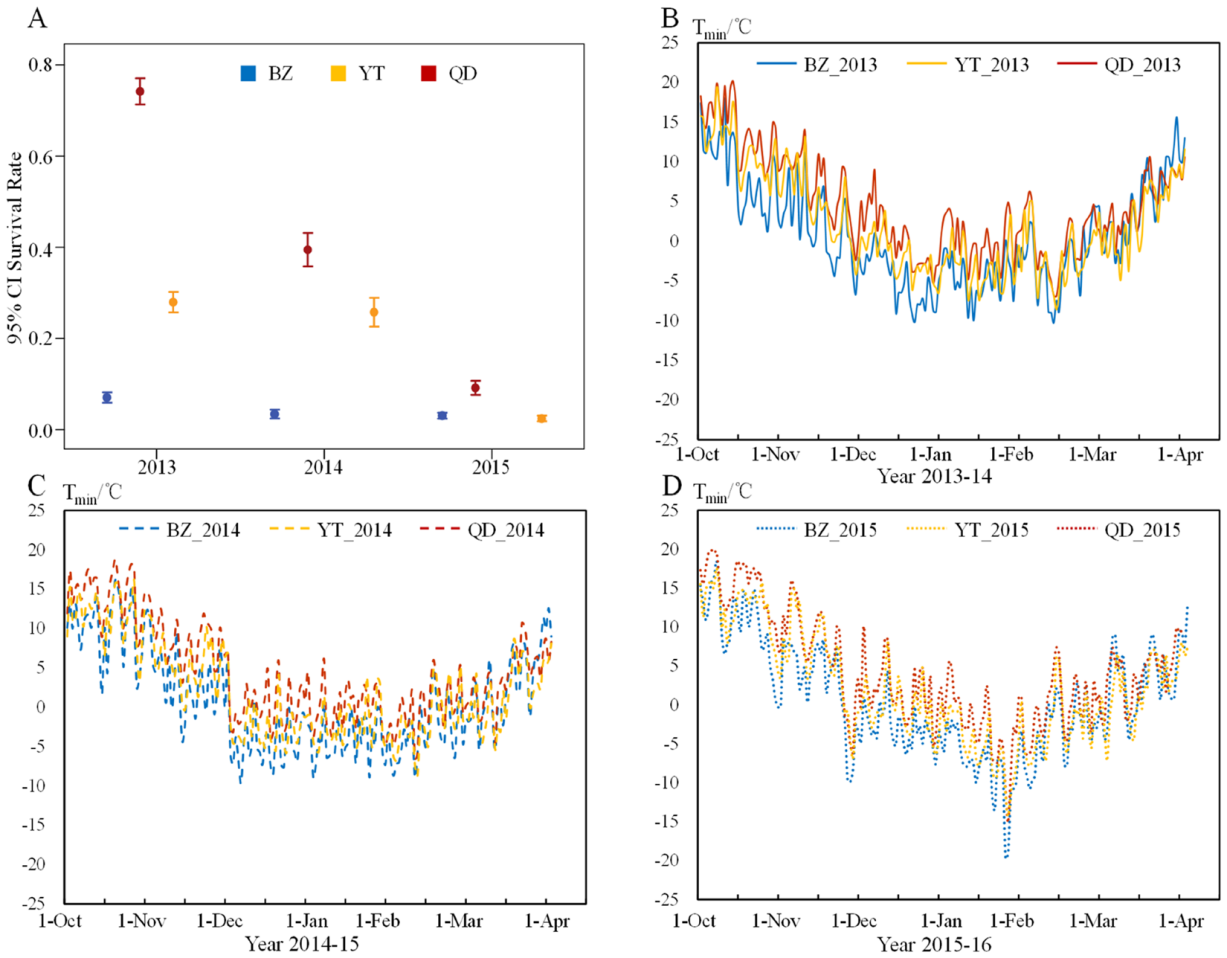
Mean survival rate (SR) of 672 pea accessions at three locations and three growing seasons (**A**) and daily lowest temperature records at each experiment (**B**–**D**). Each growing season is drawn in a separate graph and the three locations are represented with different colors: BZ, blue; YT, yellow; and QD, red.

Frost tolerance was evaluated as survival rate (SR) for 672 diverse pea germplasms in three locations and three growing seasons under field condition (Table 1 and Figure 1). It was observed that the SR values ranged from 0.03 (BZ_2015 and YT_2015) to 0.73 (QD_2013) (Table 1). There were obvious variations among the three locations as well as among growing seasons. Among the three locations, QD had the highest SR value, YT, the second, and BZ, the lowest. Among the three growing seasons, the winter in 2013 had the highest SR values, 2014, the second and 2015, the lowest. (Figure 1A). On the other hand, the fluctuation curves of the lowest temperature (T_min_) at different locations and in different growing seasons exhibited similar trends of the SR values. Namely, the T_min_ at QD is the highest, YT, the second and BZ, the lowest. For different growing seasons, the winter in 2015 had a much lower T_min_ and SR values at all three locations than the other two growing seasons (Figure 1B, C, and D). It is obvious that the winter survival of pea is closely related to the temperature changes.

**Table 1:** Summary of the analysis of variance (ANOVA) of frost tolerance in pea across Binzhou, Yantai and Qingdao, Shandong province, China from 2013 to 2016.

Trait	Year	Location	SR		*F* value of effects						
			Mean	SD	G	Y	L	G × Y	G × L	Y × L	G × Y × L
SR	2013	BZ	0.07	0.15	21.9***						
	2014	BZ	0.04	0.13	24.7***						
	2015	BZ	0.03	0.09	20.1***						
	2013	YT	0.29	0.32	52.9***						
	2014	YT	0.23	0.29	28.8***						
	2015	YT	0.03	0.08	8.9***						
	2013	QD	0.73	0.28	6.2**						
	2014	QD	0.37	0.34	93.7***						
	2015	QD	0.09	0.21	69.0***						
	2013–2015	BZ	0.05	0.13	39.1***	28.5***		5.9***			
	2013–2015	YT	0.18	0.38	56.5***	299.4***		23.5***			
	2013–2015	QD	0.40	0.28	86.2***	883.6***		26.1***			
	2013	BZ + YT + QD	0.36	0.38	64.9***		873.9***		12.7***		
	2014	BZ + YT + QD	0.21	0.30	76.9***		499.8***		35.3***		
	2015	BZ + YT + QD	0.05	0.14	62.9***		145.7***		44.9***		
	2013-2015	BZ + YT + QD	0.21	0.32	267.3***	805.1***	1171.5***	15.7***	22.1***	216.5***	17.7***

SR, survival rate; BZ, YT and QD, Binzhou, Yantai and Qingdao; G, genotype; Y, year; L, location. *P < 0.05; **P < 0.01; ***P < 0.001.

To detect the effect of variance components for SR, ANOVA were performed using a mixed-effect model. Results show that all the effects significantly affected SR at *p* = 0.001 level in all datasets except that genotypes affect SR in BZ_2013 at *p* = 0.01 level (Table 1). In combination of year and location, location exhibited the maximum effect on the variation of SR, followed by year, and then genotype. This observation demonstrates the necessity of multi-location and multi-year experiments for the evaluation of frost tolerance in pea.

**Figure 2:**
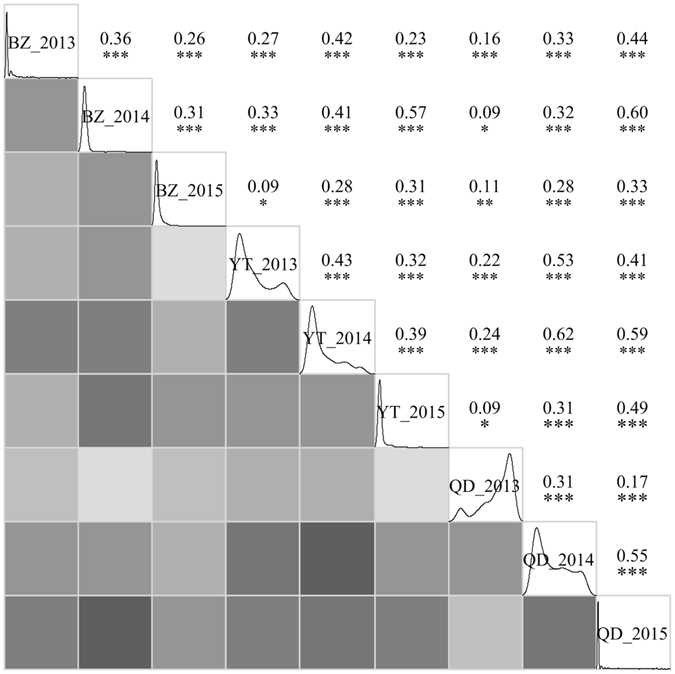
Pairwise correlations of SR between nine different field experiments at three locations and in three growing seasons. The density curves of SR were showed in the diagonal panels, and the correlations were showed both in shade map in the lower left panels and correlation matrices with significance in the upper right panels. The darker the colors are in the shade map, the stronger the relationships between different experiments. *P < 0.05; **P < 0.01; ***P < 0.001.

In addition, pairwise correlation analysis of SR was conducted for different field experiments. The correlation coefficients range from 0.09 (BZ_2014&QD_2013, BZ_2015&YT_2013 and YT_2015&QD_2013) to 0.62 (YT_2014&QD_2014) with an average of 0.34. Although all the experiments were significantly correlated with each other, the correlation coefficients between QD_2013 and other datasets are relatively low. Besides, similar trends of distributions for SR were observed in different experiments except for QD_2013 with a much higher SR than the rest (Figure 2).

A total of 16 accessions survived in all nine field experiments (three locations and three growing seasons and the lowest temperature record was −18 °C at BZ in 2015–16 growing season) (Table 2). These accessions were considered as the most winter-hardy germplasms, of which ten were from China, six from European countries. The mean SR for these germplasms was 0.57 with a range from 0.41 to 0.75. It is believed that these winter-hardy germplasms will be valuable genetic resources for winter-hardy pea cultivar breeding.

**Table 2:** Sixteen winter-hardy accessions survived in all nine field experiments for frost tolerance across Binzhou, Yantai and Qingdao, Shandong province, China from 2013 to 2016.

No.	Accession No.	Origin	Variety name	Mean of SR
1	G0000535	Henan, China	DA BO GE HUI	0.62
2	G0002295	Henan, China		0.75
3	G0002704	Jiangsu, China	QI DONG CAO WAN DOU	0.57
4	G0003468	Greece	K-129	0.50
5	G0003565	Anhui, China	921329-1	0.66
6	G0003646	Sichuan, China	MA WAN DOU	0.48
7	G0003860	Shaanxi, China	SHAN WAN DOU 8	0.55
8	G0003863	Shaanxi, China	SHAN WAN DOU 11	0.66
9	G0003864	Shaanxi, China	SHAN WAN DOU 12	0.62
10	G0003865	Shaanxi, China	SHAN WAN DOU 13	0.45
11	G0005805	Chongqing, China		0.46
12	PI 639980	Bulgaria		0.41
13	PI 269818	England		0.75
14	PI 272217	Germany		0.54
15	PI 324702	Hungary		0.48
16	PI 324703	Hungary		0.62

### Genetic variability

We genotyped 672 diverse pea accessions with 267 polymorphic SSR markers and detected a significant level of polymorphism. The number of alleles revealed by each primer pair varied from two to ten with an average of four. The average of gene diversity index and polymorphic information content (PIC) were 0.53 (ranging from 0.06 to 0.86) and 0.46 (ranging from 0.06 to 0.84), respectively.

**Figure 3:**
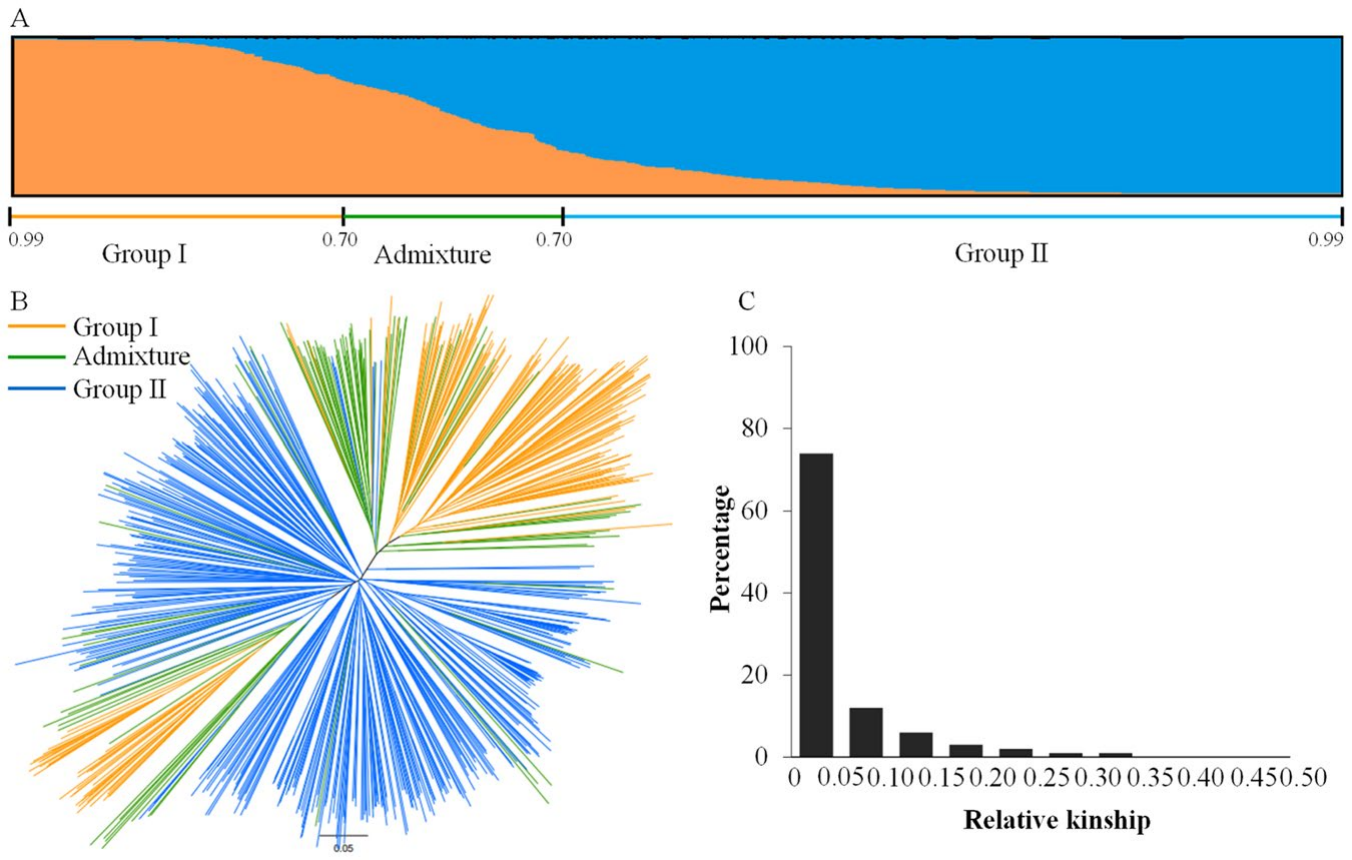
Population genetic structure (**A**,**B**) and relative kinship (**C**) of the materials. (**A**) Result of STRUCTURE analysis; (**B**) Result of NJ analysis with colored branches based on STRUCTURE analysis; and (**C**) Distribution of the kinship coefficient.

Population structure analysis was performed to avoid false association between genotype and phenotype due to the difference of allele frequency within subpopulations. Based on structure analysis, the Ln P(D) value of different K increased from 1 to 10, with delta K showing a peak at K = 2 (Fig. S1, Supporting information), which implied two sub-populations with some admixture (with q values of less than 0.7) in the collections (Figure 3A). The three subpopulations group I, group II and admixture represent 26%, 59% and 15% of the total entries, respectively (Table S1, Supplementary Information). We then investigated the geographic distribution of the genetic groups and found that group I was mainly from China (81%) and group II mainly distributed in countries outside of China (85%). It can be inferred that the genetic background of Chinese pea germplasms was significantly different from those of foreign countries.

The neighbor-joining (NJ) cluster analysis showed a similar results to structure analysis. Two clusters were identified and one is mainly containing group I and the other mainly containing group II (Figure 3B).

The kinship analysis revealed that 96% of the genotypes were detected to have little or no relationship (the kinship coefficient <0.2) suggesting that the majority of the samples are genetically diverse (Figure 3C). The relative kinship coefficient was used as K matrix in further association analysis.

### Marker-trait association analysis of frost tolerance

**Figure 4:**
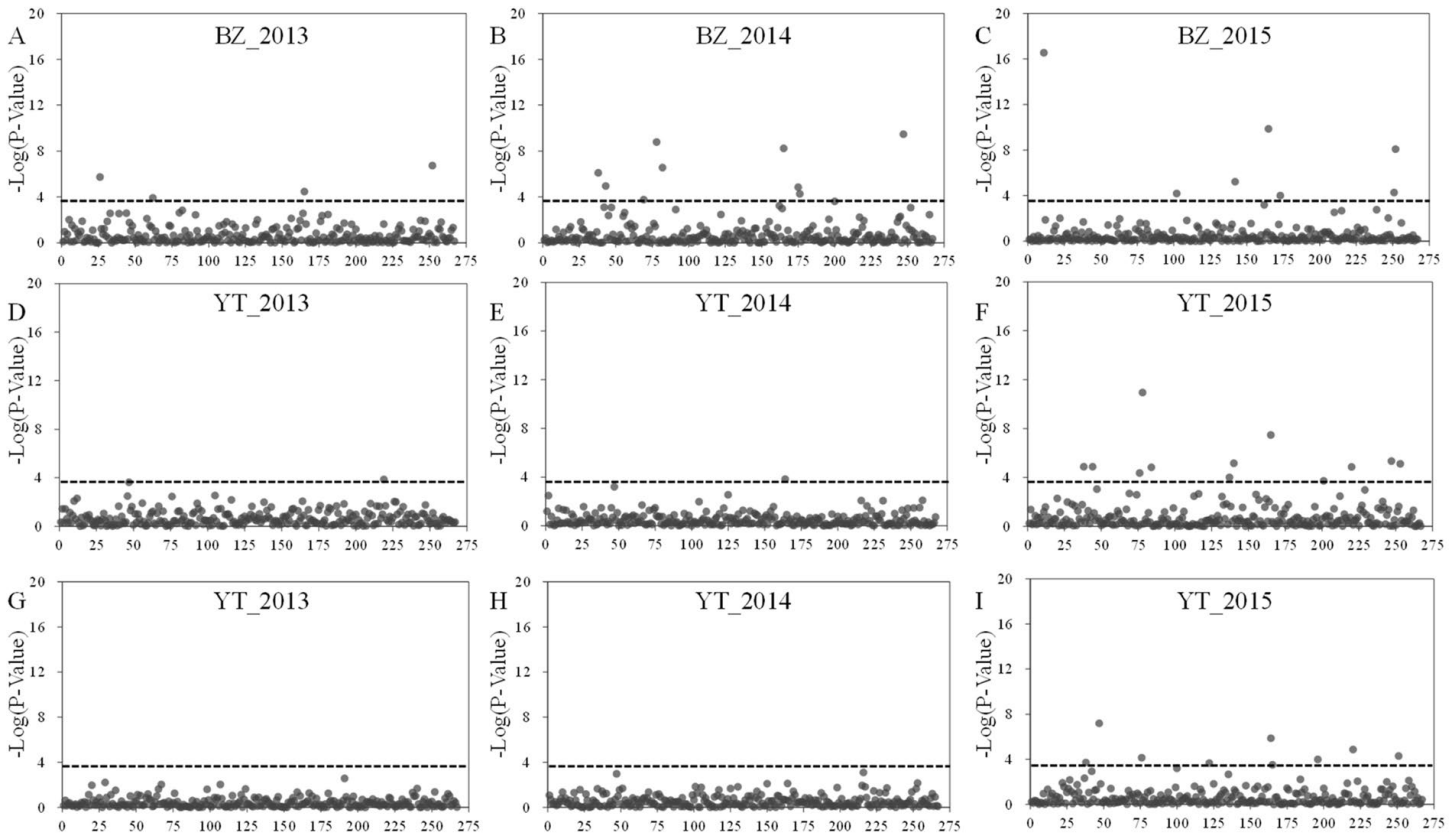
Manhattan plot of –Log_10_ *P* values of the marker-trait association for frost tolerance in pea with nine different trait datasets at three locations and three growing seasons using mixed linear model (MLM + Q + K). Threshold after sequential Bonferroni correction at 3.7 was indicated with dashed lines.

Association analysis was conducted using the genotype data and nine phenotype data-sets obtained in different field trails with two models including GLM + Q and MLM + Q + K. Under a strict threshold of 2 × 10^−4^ at a significance level of 0.05 after Bonferroni correction, a total of 58 and 24 markers were detected to have association with the frost tolerance in GLM + Q and MLM + Q + K (Figure 4), respectively, and 22 markers were present in both models (Tables S2 and S3, Supplementary Information). Comparing to GLM + Q, the significant trait-marker associations detected in MLM + Q + K decreased by nearly 60%. In addition, there are 21 and eight markers were repeatedly detected in at least two different environments in both models, respectively, and seven markers were common in both models (Table S2, Supplementary Information). These seven markers were revealed to have the most reliable associations with frost tolerance in pea (Table 3). Among the seven markers, the lowest mean of *P*-value and the largest r^2^ were observed in the marker 24142, while the marker EST723 has the maximum associations in different environments (three repetitions in GLM + Q and four repetitions in MLM + Q + K) with the second-lowest mean of *P*-value.

**Table 3:** Summary of significant marker-trait associations for frost tolerance repeatedly detected in different experiments with two models.

No.	Markers	LG	GLM + Q				MLM + Q + K			
			Repetitions	Environments	*P* value	r^2^	Repetitions	Environments	*P* value	r^2^
1	21796	LG-I	2	BZ_2015	5.3E-05	0.05	2	BZ_2015	5.8E-05	0.05
				QD_2015	1.4E-06	0.06		QD_2015	4.8E-05	0.05
2	23521	LGVII	3	YT_2013	3.9E-07	0.08	2	YT_2015	3.1E-05	0.08
				YT_2015	4.8E-06	0.08		QD_2015	5.7E-05	0.08
				QD_2015	6.7E-06	0.07				
3	24142	LGVII	2	BZ_2014	2.6E-12	0.11	2	BZ_2014	5.8E-10	0.11
				YT_2015	7.7E-11	0.10		YT_2015	6.4E-12	0.13
4	24398	LG-I	4	BZ_2013	1.8E-09	0.11	2	BZ_2013	1.5E-05	0.09
				BZ_2014	8.3E-06	0.08		BZ_2015	1.2E-08	0.12
				BZ_2015	1.3E-12	0.14				
				YT_2014	1.9E-04	0.07				
5	27068	LG-H	2	BZ_2014	3.1E-11	0.11	2	BZ_2014	2.1E-10	0.11
				YT_2015	2.0E-06	0.08		YT_2015	5.4E-06	0.08
6	EST723	LG-C	3	BZ_2014	2.4E-08	0.06	4	BZ_2013	2.8E-06	0.05
				BZ_2015	4.3E-10	0.07		BZ_2014	7.9E-09	0.06
				YT_2015	7.4E-08	0.06		BZ_2015	1.5E-10	0.08
								YT_2015	2.8E-08	0.06
7	EST1109	LGVI	2	BZ_2014	5.8E-06	0.05	3	BZ_2014	7.1E-07	0.06
				QD_2015	9.4E-05	0.04		QD_2015	1.3E-04	0.04
								YT_2015	1.4E-05	0.05

## Discussion

Frost stress is one of the major abiotic factors affecting legumes^13, 14^ and most production of pea in China are winter types^17^. One of the breeding objectives is incorporating frost tolerance into elite cultivars, which will help stabilize yield and expand the production areas northward to satisfy increasing demands of pea in China. This study evaluated to 672 diverse pea accessions including germplasm collections in China and from abroad plus a set of previously reported cold tolerant germplasm^22^ at three locations and in three growing seasons between 2013 and 2016. Our results showed that the winter survival was varied greatly among locations and growing seasons. The survival rate ranged from 0.03 to 0.73 with an average of 0.21 (Table 1). The lowest and highest winter survival were observed in Binzhou (the northernmost location with the lowest temperature) and Qingdao (the southernmost location with the highest temperature) of the three locations, while they were also observed in 2015 as the coldest winter and in 2013 with the warmest winter of the three growing seasons, respectively (Figure 1). It is suggested that the winter survival of pea is closely related to the temperature change of environment under field conditions. Winter survival is a complex trait affected by various abiotic stresses such as freezing temperature, soil moisture, snow cover and so on^23, 36, 37^. Previous studies in several crop species under controlled experiments revealed that frost tolerance is a principal cause of winter hardiness^38, 39^. Our results are consistent with the above conclusion and demonstrated the relevance between winter hardiness and frost tolerance under field conditions.

A considerable problem in field screening for frost tolerance is the variable environmental factors and the frost stress may not occur in the test year or may be so severe that all plants in test would be killed^15^. In an early study of field screening for winter hardiness peas in two locations and two growing seasons in the United States, 14 lines of *P. sativum* and four lines of *P. arvense* were screened as winter-hardy genotypes under field at Moscow, Idaho, while only winter and spring pea lines were separated with the protection of snow cover at Bozeman, Montata^23^. In the present study, three different latitudes of locations for field trails were set in the traditional spring-sown area in China with chilly winter to screen the winter hardy germplasms in three consecutive growing seasons. Significant correlations and distribution trends for winter survival were observed in all the experiments except QD_2013 (Figure 2), which may be due to the warm winter at Qingdao in 2013 (Figure 1B). In addition, 16 accessions were identified as the most winter-hardy germplasms for their ability to survive in all nine field experiments. The overall mean of SR for these 16 accessions was 0.53 and the individual accession mean ranged from 0.41 to 0.75. It is worth nothing that one of the winter-hardy accessions PI 269818 from England was previously reported as winter-hardy in winter hardy evaluation study in the USA more than 30 years ago^23^. It has been proved that multi-location and multi-year experiments can be mutual verification and provide more robust phenotyping data for further studies^16, 20, 34^. Therefore, these winter-hardy germplasms screened in this study can be used in future MAS breeding programs.

Association analysis has been performed in pea emphasizing on seed nutrients, agronomic and quality traits with different markers^33–35^. This study is the first genome-wide association analysis focused on frost tolerance in pea. We used 672 diverse pea accessions, and evaluated them in multi-location and multi-year field experiments; and generated genotyping data with 267 polymorphic SSR markers. In the association analysis, spurious associations arising from population structure should be avoid^40^. Population structure analysis detected two genetic groups with some admixtures and distinguished pea germplasms of China from those of other countries, which is similar with the result of NJ cluster analysis (Figure 3). Genetic distinction was also observed between the Chinese *P. sativum* germplasm and the global gene pool sourced outside China in a previous study to compare the genetic diversity of *Pisum* collections with a wider genetic background^41^. However, except for population structure, familial relatedness can also cause false positives in association analysis^42^. Kinship analysis revealed that most of the germplasms (96%) used in the present study showed little or no relationship between each other (Figure 3C). In present study, both GLM + Q model utilized only population structure and MLM + Q + K model incorporating population structure and relative kinship were applied in association analysis. Fifty-eight and 24 markers were identified to be significantly associated with frost tolerance in pea in GLM + Q and MLM + Q + K, respectively (Tables S2 and S3, Supplementary Information). The scope of candidate markers were narrowed down using MLM + Q + K model (Figure 4) and such reduction is consistent with other association studies^35, 42^. It is suggested that the inclusion of population structure and relative kinship in the present study had taken a full account of the possible biased effects and make the association analysis more accurately^42^.

Associated markers repeatedly detected in two or more different environments are considered more reliable than those only present in a single environment^34^. Seven such markers were identified in both models in present study (Table 3). Based on the genetic linkage map constructed in our previous study^43^, four of these markers are located in three linkage groups, LGI, VI and VII (two markers) (Table 3). Previous QTL mapping studies have identified several QTLs related to frost tolerance in pea on different genomic regions including LGI, III, V, VI and VII and some of them are related to various traits involved in frost tolerance^16, 44, 45^. A major QTL associated with freezing stress on LGIII co-localizes with the flowering gene *Hr*, which can delay flowering until long days to avoid the risk of frost damage^45^. QTLs on LGVI related to raffinose metabolism and RuBisCO activity showed a potential role in cold acclimation of pea through maintaining photosynthesis and accumulating protective sugars^44^. A QTL related to freezing damage on LGVI of *P. sativum* is found to be corresponding to a major QTL for freezing tolerance on chromosome 6 in *Medicago truncatula* ^46^. Previous studies also showed that frost tolerance is related to glycine degradation pathway, jasmonate pathway, photosynthesis, production of cryoprotectant molecules, proteins related to soluble sugar synthesis and antioxidant processes through the chloroplast, structural alteration in cell wall pectins and so on^47–49^. In present study, the marker EST1109 on LGVI significantly associated with frost tolerance is located within a functional gene that has a high homology with a gene encoding mannosyl-oligosaccharide 1, 2-alpha-mannosidase in *M. truncatula* ^50^. The locus affecting frost tolerance is discovered in this study and how this gene works awaits further investigation. It has been reported that alpha-mannosidases were involved in N-glycan processing and play a role in the development of root and the biosynthesis of cell wall in *Arabidopsis* ^51^. Recently, mannosidases were found to be associated with N-glycan degradation of glycoproteins in response to chilling stress in *Arabidopsis* ^52^. Considering that the mannosidases are involved in endoplasmic reticulum associated degradation of misfolded glycoproteins^53, 54^, it is speculated that mannosidases are involved in a general plant chilling stress response pathway^52^. In conclusion, the results of the present study suggested a novel mechanism involved in frost tolerance in pea and provided helpful references on the general chilling stress response pathway in plant.

## Methods

### Plant materials

A total of 672 diverse pea germplasms accessions with the source region covering 52 countries in the world were in the present study, of which 145 were from the pea core collection in China, 272 previously identified as cold tolerant germplasms^22^ were from the National Crop Gene Bank, located in the Institute of Crop Sciences, Chinese Academy of Agricultural Sciences, Beijing, China and 255 were from the pea core collection of the United States Department of Agriculture (USDA) Agricultural Research Service, Plant Germplasm Introduction and Testing Research Unit, Pullman, WA, USA. The detailed information is provided in Table S4 (Supplementary Information).

### Phenotyping and statistical analysis

Traditionally, the pea production in China was divided into Autumn-sown and Spring-sown regions. The field evaluation for winter hardy were carried out at three locations in the Spring-sown region in Shandong province, China. Namely, Binzhou (BZ, 37.4°N/117.9°E), Yantai (YT, Fushan (37.3°N/121.3°E) for 2013 and Laiyang (36.9°N/120.7°E) for 2014 and 2015) and Qingdao (QD, 36.2°N/120.4°E). The natural chilly winter conditions were ideal for field evaluation of winter-hardy germplasms during the growing seasons between 2013 and 2016. For field evaluation, 25 seeds of each accession were sown in a row in early October at each of the above three locations, the number of seedling emergence was recorded one and a half month after sowing before winter. After winter, the number of survival plants was counted in late March or early April. Finally, the frost tolerance were evaluated as survival rate (SR) calculating with the following formula:

$${\rm{SR}}={\rm{the}}\,{\rm{number}}\,{\rm{of}}\,{\rm{survival}}\,{\rm{plants}}/{\rm{the}}\,{\rm{number}}\,{\rm{of}}\,{\rm{seedling}}\,{\rm{emergence}}$$

The distributions of SR and pairwise correlations of nine different field trails were calculated using R software (R Development Core Team 2015). In addition, analysis of variance (ANOVA) for SR were analyzed using a mixed-effect model method in SAS^®^ 9.3 (SAS Institute Inc., Cary, North Carolina, USA) to detect the effect of genotypes, genotypes × years, genotypes × locations, years × locations and genotypes × years × locations, (Table 1). In the mix-effect model, SR was considered as dependent variables. Genotypes, locations and years were chosen as fixed effects, while replications were set as random effects.

Furthermore, the historical weather data at each location within each year were downloaded from the weather website (http://rp5.ru/Weather_in_the_world) and the change curve of the daily lowest temperature were depicted to illustrate the relationship between SR with the temperature.

### Genotyping and genetic diversity analysis

Tissue samples were collected from the tender leaves of each single plant and freeze dried in liquid nitrogen. Genomic DNA was isolated using a modified CTAB method^55^ and the concentration of DNA was determined by NanoDrop 2000c (Thermo Fisher Scientific Inc., Wilmington, Delaware, USA). After that, the DNA solution was diluted into working solution with concentration about 20–30 ng/ul, which will be used in further experiments.

A genome-wide scanning was conducted to these materials using 400 SSR markers developed by our laboratory and 267 polymorphic SSR markers were used in further analysis^56^. PCR amplification was performed in 10 ul reaction volume containing 50 ng genomic DNA, 1 ul 10*buffer, 0.2 ul dNTP (10 mmol L^−1^ each), 1 ul primer (2 umolL^−1^ each) and 0.4 U Taq DNA polymerase. PCR products were separated by 8% non-denaturing polyacrylamide gel electrophoresis (PAGE) and visualized by 0.1% silver nitrate staining.

Genotype of each accession was digital coded according to the amplified fragment length of PCR products and genetic diversity for each locus including the number of alleles, gene diversity, polymorphic information content (PIC) were calculated using PowerMarker 3.25^57^.

### Population genetic structure

Presence of population genetic structure can result in spurious associations between markers and phenotype due to the difference of allele frequency within subpopulations^58, 59^. To solve this problem, Pritchard, *et al*.^40^ developed a statistically valid method to estimate the details of population structure and integrate the information into association analysis. Therefore, inference of population genetic structure was performed using a Bayesian clustering analysis implemented in the software STRUCTRUE 2.3.3^40, 60^ to assign individuals to putative populations (k). The program was run with genotype data for k values from 1 to 10, with 20000 burn-in iterations followed by 100000 MCMC (Markov chain Monte Carlo) iterations for accurate parameter estimates. Ten independent runs for each k with correlated allele frequencies using an admixture model were performed to verify the consistency of the results. A statistic Delta K (▵K) following the procedure described by Evanno, *et al*.^61^ was applied to infer the upper-most hierarchical level of the population structure. The Q matrix of posterior probability within the best-fit subpopulations generated by the population genetic structure analysis was used as covariates in further association analysis.

Cluster analysis was also performed using neighbor-joining (NJ) method based on the allele-sharing distance by PowerMarker 3.25^57^ and displayed with Fig Tree 1.3.1 (available online http://tree.bio.ed.ac.uk/software/figtree/) to infer evolutionary relationship among genotypes.

### Association analysis

Association analysis between molecular markers and various phenotypic datasets of frost tolerance was conducted using TASSEL 3.01^62^. Markers with more than 20% missing data were removed and 259 markers were used for further association analysis. Firstly, a general linear model (GLM) in presence of population genetic structure were performed with Q matrix derived from structure analysis as covariates (GLM + Q). Secondly, a mixed linear model (MLM) incorporating a finer scale relative kinship matrix (K) and population genetic structure (Q) was applied to do the association analysis (MLM + Q + K), which improves statistical power compared to “Q” only^42^. In the MLM model, population structure and marker effect were treated as fixed effect, while individuals and residual were fit as random effects. Kinship coefficient was calculated with the distance estimation based on Loiselle, *et al*.^63^ implemented in the software SPAGeDi^64^. To control the family-wise error rate (FWER), a strict threshold of 2 × 10^−4^ at a significance level of 0.05 was chosen after Bonferroni correction. Results of marker-trait association were illustrated with a Manhattan plot (Figure 4).

## Electronic supplementary material

Supplementary Information

## References

[1] Ellis THN, Hofer JMI, Timmerman-Vaughan GM, Coyne CJ, Hellens RP (2011). Mendel, 150 years on. Trends Plant Sci..

[2] Santalla M, Amurrio JM, De Ron AM (2001). Food and feed potential breeding value of green, dry and vegetable pea germplasm. Can. J. Plant. Sci..

[3] Tian SJ, Kyle WSA, Small DM (1999). Pilot scale isolation of proteins from field peas (*Pisum sativum* L.) for use as food ingredients. Int. J. Food Sci. Tech..

[4] Phillips DA (1980). Efficiency of symbiotic nitrogen fixation in legumes. Annu. Rev. Plant Physiol..

[5] Smýkal P (2012). Pea (*Pisum sativum* L.) in the genomic era. Agronomy.

[6] FAOSTAT. http://www.fao.org/faostat/en/#data (2016).

[7] Agrawal AA, Conner JK, Stinchcombe JR (2004). Evolution of plant resistance and tolerance to frost damage. Ecol. Lett..

[8] Oakley CG, Ågren J, Atchison RA, Schemske DW (2014). QTL mapping of freezing tolerance: links to fitness and adaptive trade-offs. Mol. Ecol..

[9] Levitt, J. *Responses of plants to environmental stress: chilling, freezing and high temperature stresses* (Academic Press, New York, 1980).

[10] Xin Z, Browse J (2000). Cold comfort farm: the acclimation of plants to freezing temperatures. Plant Cell Environ..

[11] Chen LJ (2014). An overview of cold resistance in plants. J. Agron. Crop Sci..

[12] Menon M, Barnes WJ, Olson MS (2015). Population genetics of freeze tolerance among natural populations of *Populus balsamifera* across the growing season. New Phytol..

[13] Badaruddin M, Meyer DW (2001). Factors modifying frost tolerance of legume species. Crop Sci..

[14] Maqbool A, Shafiq S, Lake L (2010). Radiant frost tolerance in pulse crops-a review. Euphytica.

[15] Stoddard FL (2006). Screening techniques and sources of resistance to abiotic stresses in cool-season food legumes. Euphytica.

[16] Klein A (2014). QTL analysis of frost damage in pea suggests different mechanisms involved in frost tolerance. Theor. Appl. Genet..

[17] Li L (2017). Food legume production in China. The Crop J..

[18] Meyer DW, Badaruddin M (2001). Frost tolerance of ten seedling legume species at four growth stages. Crop Sci..

[19] Shafiq S, Mather DE, Ahmad M, Paull JG (2012). Variation in tolerance to radiant frost at reproductive stages in field pea germplasm. Euphytica.

[20] Homer A, Sahin M, Kucukozdemir U (2016). Evaluation of Pea (*Pisum sativum* L.) germplasm for winter hardiness in central Anatolia, Turkey, using field and controlled environment. Czech J. Genet. Plant. Breed..

[21] Mikic A (2011). Achievements in breeding autumn-sown annual legumes for temperate regions with emphasis on the continental Balkans. Euphytica.

[22] Zhang X (2016). Large-scale evaluation of pea (*Pisum sativum* L.) germplasm for cold tolerance in the field during winter in Qingdao. The Crop J..

[23] Auld DL, Ditterline RL, Murray GA, Swensen JB (1983). Screening peas for winterhardiness under field and laboratory conditions. Crop Sci..

[24] Annicchiarico P, Iannucci A (2008). Adaptation strategy, germplasm type and adaptive traits for field pea improvement in Italy based on variety responses across climatically contrasting environments. Field Crops Res..

[25] McPhee KE, Chen CC, Wichman DM, Muehlbauer FJ (2007). Registration of ‘Windham’ winter feed pea. J. Plant. Reg..

[26] McPhee KE, Muehlbauer FJ (2007). Registration of ‘Specter’ winter feed pea. J. Plant. Reg..

[27] Rafalski JA (2010). Association genetics in crop improvement. Curr. Opin. Plant Biol..

[28] Hamblin MT, Buckler ES, Jannink JL (2011). Population genetics of genomics-based crop improvement methods. Trends Genet..

[29] Abdurakhmonov I, Abdukarimov A (2008). Application of association mapping to understanding the genetic diversity of plant germplasm resources. Int. J. Plant Genomics.

[30] Thornsberry JM (2001). Dwarf8 polymorphisms associate with variation in flowering time. Nat. Genet..

[31] Zhou ZK (2015). Resequencing 302 wild and cultivated accessions identifies genes related to domestication and improvement in soybean. Nat. Biotechnol..

[32] Liu Y (2015). Genome-wide association study of 29 morphological traits in *Aegilops tauschii*. Scientific Reports.

[33] Cheng P (2015). Association mapping of agronomic and quality traits in USDA pea single-plant collection. Mol. Breed..

[34] Diapari M, Sindhu A, Warkentin TD, Bett K, Tar’an B (2015). Population structure and marker-trait association studies of iron, zinc and selenium concentrations in seed of field pea (*Pisum sativum* L.). Mol. Breed..

[35] Kwon SJ (2012). Genetic diversity, population structure and genome-wide marker-trait association analysis emphasizing seed nutrients of the USDA pea (*Pisum sativum* L.) core collection. Genes & Genomics.

[36] Torres AM (2010). Marker-assisted selection in faba bean (*Vicia faba* L.). Field Crops Res..

[37] Tumino G (2016). Population structure and genome-wide association analysis for frost tolerance in oat using continuous SNP array signal intensity ratios. Theor. Appl. Genet..

[38] Arbaoui M, Balko C, Link W (2008). Study of faba bean (*Vicia faba* L.) winter-hardiness and development of screening methods. Field Crops Res..

[39] Link W, Balko C, Stoddard FL (2010). Winter hardiness in faba bean: Physiology and breeding. Field Crops Res..

[40] Pritchard JK, Stephens M, Rosenberg NA, Donnelly P (2000). Association mapping in structured populations. Am. J. Hum. Genet..

[41] Zong XX (2009). Analysis of a diverse global *Pisum* sp collection and comparison to a Chinese local *P. sativum* collection with microsatellite markers. Theor. Appl. Genet..

[42] Yu JM (2006). A unified mixed-model method for association mapping that accounts for multiple levels of relatedness. Nat. Genet..

[43] Sun X (2014). SSR genetic linkage map construction of pea (*Pisum sativum* L.) based on Chinese native varieties. The Crop J..

[44] Dumont E (2009). Association of sugar content QTL and PQL with physiological traits relevant to frost damage resistance in pea under field and controlled conditions. Theor. Appl. Genet..

[45] Lejeune-Henaut I (2008). The flowering locus *Hr* colocalizes with a major QTL affecting winter frost tolerance in *Pisum sativum* L. Theor. Appl. Genet..

[46] Tayeh N (2013). A high-density genetic map of the *Medicago truncatula* major freezing tolerance QTL on chromosome 6 reveals colinearity with a QTL related to freezing damage on *Pisum sativum* linkage group VI. Mol. Breed..

[47] Baldwin L (2014). Structural alteration of cell wall pectins accompanies pea development in response to cold. Phytochemistry.

[48] Grimaud F (2013). Exploring chloroplastic changes related to chilling and freezing tolerance during cold acclimation of pea (*Pisum sativum* L.). J. Proteomics.

[49] Legrand S (2013). Combining gene expression and genetic analyses to identify candidate genes involved in cold responses in pea. J. Plant Physiol..

[50] Tang H (2014). An improved genome release (version Mt4.0) for the model legume *Medicago truncatula*. BMC Genomics.

[51] Liebminger E (2009). Class I alpha-mannosidases are required for N-glycan processing and root development in *Arabidopsis thaliana*. Plant Cell.

[52] Ma J (2016). Endoplasmic reticulum-associated N-glycan degradation of cold-upregulated glycoproteins in response to chilling stress in *Arabidopsis*. New Phytol..

[53] Huttner S (2014). *Arabidopsis* Class I alpha-mannosidases MNS4 and MNS5 are involved in endoplasmic reticulum-associated degradation of misfolded glycoproteins. Plant Cell.

[54] Tokunaga F, Brostrom C, Koide T, Arvan P (2000). Endoplasmic reticulum (ER)-associated degradation of misfolded N-linked glycoproteins is suppressed upon inhibition of ER mannosidase I. J. Biol. Chem..

[55] Dellaporta SL, Wood J, Hicks JB (1983). A plant DNA minipreparation version II. Plant Mol. Biol. Rep..

[56] Yang T (2015). High-throughput development of SSR markers from pea (*Pisum sativum* L.) based on next generation sequencing of a purified Chinese commercial variety. PLoS ONE.

[57] Liu K, Muse SV (2005). PowerMarker: an integrated analysis environment for genetic marker analysis. Bioinformatics.

[58] Ewens WJ, Spielman RS (1995). The transmission disequilibrium test - history, subdivision and admixture. Am. J. Hum. Genet..

[59] Pritchard JK, Rosenberg NA (1999). Use of unlinked genetic markers to detect population stratification in association studies. Am. J. Hum. Genet..

[60] Falush D, Stephens M, Pritchard JK (2003). Inference of population structure using multilocus genotype data: Linked loci and correlated allele frequencies. Genetics.

[61] Evanno G, Regnaut S, Goudet J (2005). Detecting the number of clusters of individuals using the software STRUCTURE: a simulation study. Mol. Ecol..

[62] Bradbury PJ (2007). TASSEL: software for association mapping of complex traits in diverse samples. Bioinformatics.

[63] Loiselle BA, Sork VL, Nason J, Graham C (1995). Spatial genetic-structure of a tropical understory shrub, *Psychotria officinalis* (*Rubiaceae*). Am. J. Bot..

[64] Hardy OJ, Vekemans X (2002). SPAGEDi: a versatile computer program to analyse spatial genetic structure at the individual or population levels. Mol. Ecol. Notes.

