# Supplementary material for: Marker-trait association analysis of frost tolerance of 672 worldwide pea (Pisum sativum L.) collections
Source: Sci Rep. 2017 Jul 19;7:5919. doi: 10.1038/s41598-017-06222-y (PMC5517424; doi:10.1038/s41598-017-06222-y)
Supplement: Supplementary file 1 — Supplementary Information [file 41598_2017_6222_MOESM1_ESM.pdf]

Supplemental Information

Marker-trait association analysis of frost tolerance of 672 worldwide pea (*Pisum sativum* L.) collections

Rong Liu[1†], Li Fang[1†], Tao Yang[1†], Xiaoyan Zhang[2], Jinguo Hu[3], Hongyan Zhang[1], Wenliang Han[4], Zeke Hua[5], Junjie Hao[2], Xuxiao Zong[1*]

[1] Center for Crop Germplasm Resources / Institute of Crop Sciences, Chinese Academy of Agricultural Sciences, Beijing 100081, China

[2] Qingdao Academy of Agricultural Sciences, Qingdao 266100, Shandong, China

[3] USDA, Agricultural Research Service, Western Regional Plant Introduction Station, Washington State University, Pullman, WA 99164, USA

[4] Binzhou Academy of Agricultural Sciences, Binzhou 256600, Shandong, China

[5] Laiyang Agricultural Extension Center, Laiyang 265200, Shandong, China

[†]These authors contributed equally to this work.

*Corresponding author: Xuxiao Zong

Center for Crop Germplasm Resources / Institute of Crop Sciences, Chinese Academy of Agricultural Sciences, Beijing 100081, China

Tel: +86-10-62186651

Fax: +86-1062186651

E-mail: zongxuxiao@caas.cn

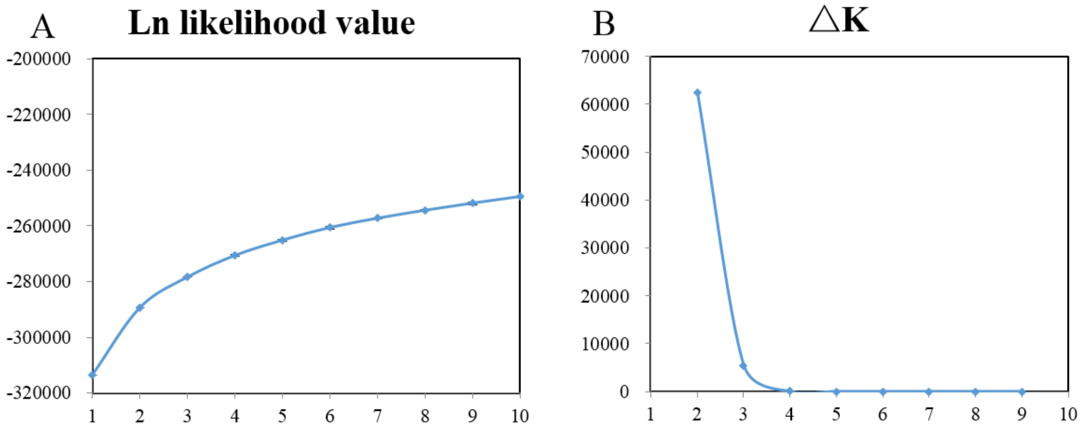

Fig. S1 Ln likelihood value (A) and ∆K (B) of STRUCTURE analysis.

Table S1 Geographic distribution of different genetic groups

Structure Group	China	other countries	unknown	Total
Group 1	81%	17%	2%	26%
Group 2	8%	85%	7%	59%
Group 3	66%	19%	14%	15%
SUM	36%	57%	7%	100%

Table S2 Summary of significant marker-trait associations for frost tolerance with two models

NO	Markers	LG	GLM+Q				MLM+Q+K			
			Repetitions	Environments	P value	R^2	Repetitions	Environments	P value	R^2
1	21796	LG-I	2	BZ_2015	5.3E-05	0.05	2	BZ_2015	5.8E-05	0.05
				QD_2015	1.4E-06	0.06		QD_2015	4.8E-05	0.05
2	23521	LGVII	3	YT_2013	3.9E-07	0.08	2	YT_2015	3.1E-05	0.08
				YT_2015	4.8E-06	0.08		QD_2015	5.7E-05	0.08
				QD_2015	6.7E-06	0.07				
3	24142	LGVII	2	BZ_2014	2.6E-12	0.11	2	BZ_2014	5.8E-10	0.11
				YT_2015	7.7E-11	0.10		YT_2015	6.4E-12	0.13
4	24398	LG-I	4	BZ_2013	1.8E-09	0.11	2	BZ_2013	1.5E-05	0.09
				BZ_2014	8.3E-06	0.08		BZ_2015	1.2E-08	0.12
				BZ_2015	1.3E-12	0.14				
				YT_2014	1.9E-04	0.07				
5	27068	LG-H	2	BZ_2014	3.1E-11	0.11	2	BZ_2014	2.1E-10	0.11
				YT_2015	2.0E-06	0.08		YT_2015	5.4E-06	0.08
6	EST723	LG-C	3	BZ_2014	2.4E-08	0.06	4	BZ_2013	2.8E-06	0.05
				BZ_2015	4.3E-10	0.07		BZ_2014	7.9E-09	0.06
				YT_2015	7.4E-08	0.06		BZ_2015	1.5E-10	0.08
								YT_2015	2.8E-08	0.06
7	EST1109	LGVI	2	BZ_2014	5.8E-06	0.05	3	BZ_2014	7.1E-07	0.06
				QD_2015	9.4E-05	0.04		QD_2015	1.3E-04	0.04
								YT_2015	1.4E-05	0.05

8	16512	LGII	2	QD_2014	7.9E-06	0.04	0			
				QD_2015	1.4E-04	0.04				
9	16524	LGIII	2	BZ_2013	1.6E-04	0.07	0			
				YT_2014	1.2E-05	0.08				
10	18013	LGVII	2	BZ_2014	2.0E-04	0.07	0			
				YT_2013	3.4E-05	0.07				
11	18339	LGI	2	YT_2013	7.7E-06	0.04	0			
				YT_2014	8.8E-05	0.04				
12	25387	LG-C	2	BZ_2014	1.9E-04	0.04	1	BZ_2014	5.9E-05	0.05
				YT_2013	7.9E-06	0.05				
13	27301	LGI	2	BZ_2015	2.5E-20	0.14	1	BZ_2015	2.0E-17	0.15
				YT_2013	3.4E-05	0.04				
14	27491	LGVI	5	BZ_2014	2.1E-05	0.04	1	QD_2015	5.6E-08	0.07
				YT_2013	3.7E-09	0.06				
				YT_2014	4.1E-05	0.04				
				QD_2014	2.2E-05	0.03				
				QD_2015	6.8E-08	0.06				
15	28108	LGVII	2	BZ_2013	8.5E-06	0.04	0			
				BZ_2014	5.8E-05	0.04				
16	28374	LG-B	3	BZ_2013	1.0E-04	0.04	0			
				BZ_2015	1.7E-04	0.04				
				YT_2015	1.9E-05	0.05				
17	28383	LG-E	2	BZ_2013	1.0E-04	0.04	1	YT_2013	1.5E-04	0.04
				YT_2013	3.1E-07	0.05				

18	28654	LGI	4	BZ_2013	1.1E-07	0.05	0			
				YT_2014	3.6E-06	0.07				
				YT_2015	6.2E-05	0.03				
				QD_2015	1.5E-04	0.02				
19	28687	LG-E	1	YT_2015	1.1E-04	0.03	2	YT_2015	1.2E-05	0.04
								QD_2015	1.3E-05	0.04
20	29331	LGVI	2	YT_2014	1.6E-05	0.04	0			
				QD_2014	1.2E-04	0.03				
21	EST625	LG-C	7	BZ_2013	7.5E-06	0.05	1	QD_2015	1.5E-06	0.06
				BZ_2014	1.3E-07	0.06				
				YT_2013	4.3E-07	0.05				
				YT_2014	4.8E-09	0.07				
				YT_2015	4.6E-08	0.07				
				QD_2014	7.3E-06	0.04				
				QD_2015	3.6E-13	0.09				
22	EST656	LG-B	3	QD_2015	1.0E-04	0.06	0			
				YT_2014	4.6E-05	0.07				
				YT_2015	1.7E-04	0.07				
23	16570	LG-G	1	YT_2013	1.9E-07	0.05	0			
24	17431	LGVII	1	QD_2015	6.5E-05	0.07	0			
25	17713	LG-G	1	YT_2015	7.6E-06	0.10	0			
26	17754	LG-G	1	YT_2014	6.1E-05	0.05	0			
27	18928	LG-A	1	QD_2014	8.3E-05	0.03	0			
28	21250	LG-B	1	YT_2014	1.8E-04	0.09	0			

29	21726	LGIII	1	QD_2015	1.2E-04	0.10	0			
30	22276	LG-B	1	YT_2015	5.0E-05	0.07	0			
31	22352	LG-B	1	YT_2015	1.6E-08	0.07	1	YT_2015	1.0E-04	0.04
32	22599	LG-B	0				1	YT_2015	5.8E-06	0.05
33	23358	LGI	1	BZ_2013	9.3E-05	0.03	0			
34	23518	LG-D	1	QD_2013	1.4E-04	0.07	0			
35	24236	LG-C	1	BZ_2015	2.7E-07	0.07	1	BZ_2015	5.7E-05	0.06
36	24547	LGVII	1	BZ_2014	2.3E-12	0.13	1	BZ_2014	1.1E-07	0.12
37	24560	LGVI	1	BZ_2014	8.2E-05	0.04	1	BZ_2014	2.2E-06	0.06
38	24588	LGVII	1	BZ_2013	4.9E-07	0.08	0			
39	24602	LGVII	1	QD_2014	4.2E-05	0.03	0			
40	24652	LG-I	1	YT_2015	4.0E-08	0.07	1	YT_2015	3.0E-06	0.06
41	25059	LG-E	1	QD_2014	3.8E-05	0.03	0			
42	25076	LG-C	1	BZ_2014	1.0E-06	0.09	1	BZ_2014		
43	25711	LGVI	1	YT_2015	8.0E-11	0.13	1	YT_2015	1.8E-05	0.12
44	25755	LG-C	1	BZ_2013	4.4E-05	0.06	0			
45	25965	LGVII	1	YT_2015	1.0E-08	0.19	1	YT_2015	1.2E-05	0.17
46	26575	LG-D	0				1	QD_2015	9.7E-05	0.05
47	27361	LGI	1	YT_2013	1.5E-06	0.05	0			
48	27583	LGVII	1	BZ_2015	4.8E-07	0.05	1	BZ_2015	2.9E-05	0.04
49	28790	LGVI	1	YT_2014	4.8E-05	0.03	0			
50	29839	LG-C	1	QD_2015	1.7E-04	0.03	0			
51	29955	LG-B	1	YT_2015	6.5E-05	0.05	0			
52	30379	LGVI	1	YT_2013	1.9E-04	0.02	0			

53	EST527	LGVII	1	YT_2015			0
54	EST734	LG-A	1	YT_2013	1.9E-04	0.05	0
55	EST878	LG-B	1	YT_2013	1.1E-06	0.05	0
56	EST921	LGI	1	YT_2015			0
57	EST956	LG-E	1	YT_2015			0
58	EST968	LGVII	1	QD_2014	1.9E-04	0.04	0
59	EST1276	LGVII	1	BZ_2014	8.5E-06	0.04	0
60	S85	LGVII	1	YT_2013	1.7E-04	0.03	0

Table S3 Primer information of the 60 frost tolerance associated SSR markers

NO	Markers	LG	Forward primer seq.	Reverse primer seq.
1	21796	LG-I	TCTTCGCTGGGAAGTTGAGT	GGAAGCGATGTCGTTTCATT
2	23521	LGVII	CGCCAATTCCTTTTCCCTAC	AGAACTCACAGGCGATGGTC
3	24142	LGVII	GCAGCCATGGTTGATTGATT	TCAAGAACATTACTTTTTCCCTCT
4	24398	LG-I	TTCGATGCATGAATGACAAA	ATCGGCGGAGACTAAGATCA
5	27068	LG-H	TTTCGGGCGTCAAATAATTC	GCCACACCTCCAAATGAGTT
6	EST723	LG-C	GGGGGTGTCTTACGTTGATG	CCCCAAAACCAGCTGAACTA
7	EST1109	LGVI	TCCGGCAAGATATTGGAAAA	GCTTGGATCGCAGGAAAATA
8	16512	LGII	TAAGCCCGACGCTTCTATTC	GTGCCTCAGTTTCCGTTTGT
9	16524	LGIII	CCAGAGGATGTGAACCAGGTA	TTCAACCAAGCTGAACCCTTA
10	18013	LGVII	TCAATTCCGAACCACCTTTC	CGGCAGAATTAGGGTTTTGA
11	18339	LGI	TGGTTGAACTGGAACGAGTG	TGAAATTGCAATGTAAGCATGA
12	25387	LG-C	GGCTCATGCATCTACCACCT	ATCCCGACGTTCACATTTTC
13	27301	LGI	TGTCGGAAATTAAGAGGTGGA	TGGAAAAGTAAGCGGTGAACA
14	27491	LGVI	TCCTAACCAACCAATAACACGAT	TTGAGGATTTCGGTGACCTC
15	28108	LGVII	CGACAATGTTGCCAGCTATC	TTTTAGGATTTTATCGACGTTTTTC
16	28374	LG-B	TCCACGGTCTTGCTATGTGT	CTGGTTGCACATCAGGGTAG
17	28383	LG-E	TCGATTGTTATTGTGTTTCCTCTC	TGAGATCAAGTGGGGGAAAA
18	28654	LGI	AGCGACGTGAATATCACAATG	GTTATCGCGGCGTGTAAATC
19	28687	LG-E	CACGGAAGGCCCTACTTACA	GTGGCGAGTAGAGCGTAAGG
20	29331	LGVI	GGGTGGACCGAATATTTCAA	CGTCACCTCTACCGAAGCTC
21	EST625	LG-C	GCTCCAATGGCTTCCTAACA	AACAAGGGGCAATCACAATC
22	EST656	LG-B	AGCAGCTCATATTCTCTGTCCA	AGCAGAAGCAGAAGCAGGAT
23	16570	LG-G	CAAACACCAACCACCACAGT	AAGGGGAGACGAAGTGGAGT
24	17431	LGVII	TTCACAATTCACCACCAATCA	CCAACGTCAGGTACGATTCA

25	17713	LG-G	AAAAAGGGGAAAGCAGGAGA	TTGACTGTGAGGCTGGTTTG
26	17754	LG-G	AGCAACGGGCAACCTTATAG	CCTTTTGTTTGGAAGCTCAA
27	18928	LG-A	TGAATGTGGAAAGGAGGAATG	AGGGTCACCACTTTGGAGAG
28	21250	LG-B	GTGCAATTTTCACACAGTGG	ACGAAGGTTGGAGCATGATT
29	21726	LGIII	GGTGATGGAGAAAAGGGTGA	TGCATGCAGTCAAATCAAAA
30	22276	LG-B	ATGCGGCATTTTGCTTTATC	TTGGTCTGCAAATCGAAACA
31	22352	LG-B	CCAACATCTTCCTCATCACCT	TGAGAGTCGCAGTCGGATAA
32	22599	LG-B	GAACATTATGGCGAAAGTTGA	TGGACAAGGCTCCTCTAAAGTT
33	23358	LGI	CCAATCTCAAGATCCATCACC	TCGGAAGCTTATCGGAGAAA
34	23518	LG-D	CAAGGACGACGACAACAACA	GTGCCGACGTTCAAGAAAAT
35	24236	LG-C	CAAACCTTCTTTATTTCCATTTCA	ACTTCTGGTCCACGCAAAAC
36	24547	LGVII	CGGCAGAATTAGGGTTTTGA	TCAATTCCGAACCACCTTTC
37	24560	LGVI	GATAAAGGCAGCGACAGAGG	AATGAAGTGCAAGCCCAAAT
38	24588	LGVII	AAATAGATGAGAAAGAGAGAGATTACG	CGCACTTCCATTCACATGAT
39	24602	LGVII	TGAGTGGGCGTGTGATTTAG	TTGCACTGTCGCATTTGAGT
40	24652	LG-I	GAGAAAGCGGCTGCTTAGAA	GCTGTCACCGAGAATGATGA
41	25059	LG-E	ATGGATTGCGGATAGCTCAA	CAGCAGTTGTTCGCAGGTAA
42	25076	LG-C	GCTTGCAAGTGTGCGTGTAT	CCAGCCAAATGCACAATAAA
43	25711	LGVI	AAGGTTTTGAAATAAATGAAGTTTG	TGAAAGCCCACTTGATCTTC
44	25755	LG-C	TTTTCCAACTAAGGTTGTTTCTTTC	CAAAAGGAGGAGGCTGAAGA
45	25965	LGVII	TGATTCGTAGACCCCACACA	AAGGTTAATGTCTTCTTTTTGAAGTT
46	26575	LG-D	GAAAATAAACAGTTGGCAACAAAA	CCACTCCAAACCCTTCAGGT
47	27361	LGI	CTGAAACGGTTTGCATTGTG	TCCAACCACTTCTTAACAACCT
48	27583	LGVII	TGCACAGAGGATGGTTCTCA	TGGATTGAGCCTCTTGTCCT
49	28790	LGVI	GCTGTGGGGGTTTAATCAGA	CCGCAATCCTTCAAGAACTC
50	29839	LG-C	GAACCTCGTTTTTGCATCCT	AATGATAGGGGTTGCCACAT

51	29955	LG-B	TCAAGTGCATTGGGAGAGACT	AAAAACCGACCCATAATCAATTT
52	30379	LGVI	TGTTGGCAGGAAACTCTTCA	AGCCACAAATTTCGTTGTGTT
53	EST527	LGVII	TTGAAGCAGTGGCAGAGTTG	TCTCAATGAAACATAAGAATGACCTT
54	EST734	LG-A	AGGCAGTGACTGAATCATCGT	AATGGCTTTGAGGCAGAGAG
55	EST878	LG-B	CGCATTTTCACTCCACACAC	CGTTCGGAACATCCAAGGT
56	EST921	LGI	AAGGGGTGATCAAGCATCAA	TTGAGGGAACATGAAGAAATCA
57	EST956	LG-E	CGAGCGTGAGACTGTGATGT	TCCACCGGTTCAACTTCAAT
58	EST968	LGVII	ACCGCTTGAACTCCAAACAA	GAAGGTAACAACGCCGAGAA
59	EST1276	LGVII	TGAAACAATAGTGCTTTGTTGAAACT	TTTTCTCGTCTGCGTGTGAC
60	S85	LGVII	TTCCAACCATGGAAGCTTTT	TTCTTCGTCGGGTACAGTGA

Table S4 Detailed information of the materials

No.	Accession No.	Origin	Variaty name/Plant ID	Mean of SR	Winter survival times	Strucure group (>70)
1	G000033	Henan, China		0.71	8	1
2	G000043	Yunnan, China		0.15	5	3
3	G0000184	Inner Mongolia, China		0.23	4	2
4	G0000202	Inner Mongolia, China	DA WAN DOU	0.19	3	1
5	G0000238	Inner Mongolia, China	HUI LV WAN DOU	0.18	2	1
6	G0000259	Inner Mongolia, China	BEN DI WAN DOU	0.57		1
7	G0000296	Inner Mongolia, China	LI MIAO WAN DOU	0.31	5	1
8	G0000314		WU MING WAN DOU ER HAO	0.15	3	3
9	G0000317		BAI WAN DOU	0.26	4	3
10	G0000400	Shaanxi, China	XING PING TOU	0.49	7	1
11	G0000408	Shaanxi, China	BAI CAI WAN DOU	0.29	6	3
12	G0000413	Shaanxi, China	BAI WAN DOU	0.34	5	1
13	G0000469	Shaanxi, China		0.44	7	1
14	G0000474	Shaanxi, China		0.62	8	1
15	G0000506	Shaanxi, China	HONG HUA CAI WAN DOU	0.54	8	1
16	G0000527	Henan, China	BAI WAN DOU	0.41	6	3
17	G0000535	Henan, China	DA BO GE HUI	0.62	9	1
18	G0000546	Sichuan, China	FEN HONG	0.24	6	3
19	G0000555	Xinjiang, China	KA JIN TA LE WAN DOU	0.16	4	3
20	G0000566	Qinghai, China	GA BAI DOU	0.06	3	2
21	G0000574	Qinghai, China	XIAO BAI DOU	0.37	8	1
22	G0000576	Qinghai, China	DA BAI DOU	0.05	1	1
23	G0000582	Qinghai, China	XIAO NIU MI DOU	0.12	3	1
24	G0000600	Qinghai, China	DA BAI DOU	0.30	3	1
25	G0000626	Qinghai, China	ZHUAN YAN BAI DOU	0.10	2	1
26	G0000629	Qinghai, China	DA BAI DOU	0.38	5	1
27	G0000636	Qinghai, China	HONG HUA BAI DOU	0.17	3	1
28	G0000643	Qinghai, China		0.31	4	1
29	G0000645	Qinghai, China	LIU SHI DOU	0.02	1	2
30	G0000658	Qinghai, China	QING WAN DOU	0.29	5	1
31	G0000671	Qinghai, China	XIAO QING WAN DOU	0.35	4	1
32	G0000685	Qinghai, China	DA QING DOU	0.21	4	1

33	G0000697	Qinghai, China	XIAO QING DOU	0.14	3	1
34	G0000752	Inner Mongolia, China		0.31	6	1
35	G0000753	Inner Mongolia, China		0.25	5	1
36	G0000754	Zhejiang, China	1924	0.13	4	2
37	G0000758	Fujian, China	HONG HUA WAN DOU	0.42	8	1
38	G0000759	Jiangxi, China	BAI HUA WAN DOU	0.17	5	1
39	G0000760	Henan, China	DENG FENG BAI	0.51	6	1
40	G0000761	Hunan, China		0.51	7	3
41	G0000763	Guangdong, China	2 HAO KANG BING BAI XI	0.30	5	1
42	G0000764	Guangdong, China	5 HAO BAI HUA XIAO TUO YE	0.16	6	2
43	G0000794	Qinghai, China		0.23		3
44	G0000880		QIU FU YI HAO	0.27	5	1
45	G0000883	Qinghai, China	CAO YUAN 7 HAO	0.13	3	3
46	G0000885		BIAN JIN WAN DOU	0.10	3	2
47	G0000891	Beijing, China	ZAO SHU XIAO WAN DOU	0.06	2	2
48	G0000894	Heilongjiang, China		0.01	1	2
49	G0000897	Shanghai, China		0.01	1	2
50	G0000917	Tibet, China	BAI WAN DOU	0.10	3	3
51	G0000924	Tibet, China		0.14	4	1
52	G0000927	Xinjiang, China	HA MI WAN DOU	0.08	3	2
53	G0000928	Taiwan, China		0.06	2	2
54	G0000931	Yunnan, China		0.07	4	2
55	G0000938	Shanghai, China	BAN GAO GAN	0.16	5	3
56	G0000941	Yunnan, China	CAI WAN DOU	0.04	3	3
57	G0000945	Tibet, China		0.21	5	2
58	G0000947	Tibet, China	QING WAN DOU	0.18	5	3
59	G0000959	Tibet, China	HEI WAN DOU	0.25	6	1
60	G0000962	Tibet, China	HEI WAN DOU	0.11	3	1
61	G0000972	Tibet, China	MA WAN DOU	0.04	1	1
62	G0000988	Tibet, China		0.08	2	1
63	G0000995	Qinghai, China		0.15	4	3
64	G0000999	Taiwan, China		0.07	3	2
65	G0001000	Taiwan, China		0.19	5	3
66	G0001002	Shanxi, China	LI MIN BAI WAN DOU	0.35	6	2
67	G0001044	Shanxi, China	DA KE WAN DOU	0.15	3	2
68	G0001117	Inner Mongolia, China	BAI WAN DOU	0.45		1
69	G0001152	Henan, China	DUAN YANG BAI WAN DOU	0.50	8	1
70	G0001246	Hubei, China	BAI XIAO WAN DOU	0.37	5	3
71	G0001314	Guangxi, China	HE LAN DOU	0.46	6	1

72	G0001380	Sichuan, China	DA BAI WAN	0.18	4	2
73	G0001464	Sichuan, China	LU WAN	0.43	5	1
74	G0001470	Sichuan, China	QING PI CAI	0.15	3	3
75	G0001484	Sichuan, China	DA BAI WAN	0.20	4	1
76	G0001546	Sichuan, China	HUANG MA WAN	0.11	3	3
77	G0001552	Chongqing, China	MA WAN	0.20	3	1
78	G0001559	Chongqing, China	DA MA WAN	0.13	4	1
79	G0001715	Guizhou, China	DA BAI WAN	0.13	3	1
80	G0001747	Yunnan, China	BAI WAN DOU	0.32	5	3
81	G0001752	Yunnan, China		0.28	4	3
82	G0001758	Yunnan, China	BAI WAN DOU	0.25	3	1
83	G0001772	Yunnan, China		0.11	3	2
84	G0001778	Yunnan, China	DA BAI WAN DOU	0.23	5	3
85	G0001794	Yunnan, China	CAI WAN DOU	0.30	4	1
86	G0001795	Yunnan, China	MA WAN DOU	0.08	2	3
87	G0001803	Yunnan, China		0.12	3	1
88	G0001809	Shaanxi, China	BAI WAN DOU	0.42	6	1
89	G0001811	Shaanxi, China	BAI WAN DOU	0.11	4	1
90	G0001812	Shaanxi, China	CAI WAN DOU	0.39	5	1
91	G0001835	Shaanxi, China	AI WAN DOU	0.40	5	2
92	G0001839	Shaanxi, China	DA BAI WAN DOU	0.26	3	2
93	G0001850	Shaanxi, China		0.37	5	1
94	G0001859	Shaanxi, China	MA CAI WAN DOU	0.24	3	2
95	G0001871	Shaanxi, China	CAI WAN DOU	0.46	8	1
96	G0001880	Shaanxi, China	CAI WAN DOU	0.54	8	3
97	G0001890	Shaanxi, China	MA WAN DOU	0.51	7	3
98	G0001910	Shaanxi, China	MA WAN DOU	0.29	6	1
99	G0001966	Gansu, China	BAI WAN DOU	0.42	8	1
100	G0002001	Gansu, China	LU WAN DOU	0.21	3	1
101	G0002036	Gansu, China	LAN HUA WAN DOU	0.28	6	1
102	G0002078	Tibet, China	WEN MA DOU	0.34	6	1
103	G0002261	Beijing, China	ZHONG WAN 4 HAO	0.27	7	1
104	G0002269	Qinghai, China	CAO YUAN 12 HAO	0.02	2	2
105	G0002275	Shanghai, China		0.02	1	2
106	G0002278	Ningxia, China		0.23	4	1
107	G0002279	Beijing, China	TIAN WAN DOU	0.18	3	2
108	G0002283	Hunan, China	BAI WAN DOU	0.21	5	2
109	G0002294	Henan, China	LI WAN DOU	0.68	8	1
110	G0002295	Henan, China		0.75	9	1
111	G0002300	Henan, China	BO GE HUI	0.27	6	1
112	G0002337	Jiangxi, China	XUE DOU	0.21	5	1
113	G0002502	Xinjiang, China		0.20	5	3
114	G0002620	Hebei, China	YUAN BAO SHAN WAN DOU	0.06	4	2

115	G0002639	Inner Mongolia, China	BAI WAN DOU	0.15	4	2
116	G0002655	Inner Mongolia, China	LU WAN DOU	0.12	4	3
117	G0002677	Inner Mongolia, China	MA LI WAN DOU	0.29	5	3
118	G0002679	Inner Mongolia, China		0.20	5	1
119	G0002686	Inner Mongolia, China	LU WAN DOU	0.43	8	1
120	G0002704	Jiangsu, China	QI DONG CAO WAN DOU	0.57	9	1
121	G0002789	Chongqing, China	SHUI HONG WAN DOU	0.09	3	3
122	G0002800	Chongqing, China	AI ZI WAN DOU	0.12	3	3
123	G0002811	Chongqing, China	MA WAN DOU	0.11	3	1
124	G0002823	Guizhou, China	HONG HUA WAN	0.40	8	3
125	G0002875	Germany	HJA 51879-AF	0.13	1	2
126	G0002997	Inner Mongolia, China	LU WAN DOU	0.06	1	3
127	G0003001	Inner Mongolia, China	LU PI WAN DOU	0.39	6	1
128	G0003044	Anhui, China		0.51		1
129	G0003045	Anhui, China		0.61	7	1
130	G0003057	Anhui, China		0.49	5	1
131	G0003058	Anhui, China		0.43	9	1
132	G0003061	Anhui, China		0.37	6	1
133	G0003083	Anhui, China	HONG WAN DOU	0.33	6	1
134	G0003090	Anhui, China	BAI WAN DOU	0.02	2	1
135	G0003091	Anhui, China	BAI WAN DOU	0.06	3	3
136	G0003093	Anhui, China	LI WAN DOU	0.32	5	1
137	G0003094	Anhui, China		0.24	5	1
138	G0003202	Hubei, China	BAI WAN DOU	0.27	5	1
139	G0003213	Hubei, China		0.38	4	1
140	G0003214	Hubei, China		0.27	5	3
141	G0003223	Hubei, China	MI WAN DOU	0.15		1
142	G0003234	Hubei, China	ZI WAN DOU	0.31	7	3
143	G0003246	Hubei, China		0.44	6	1
144	G0003261	Hubei, China	CAI WAN DOU	0.47	6	3
145	G0003267	Hubei, China	CAI WAN DOU	0.42	6	3
146	G0003268	Hubei, China	MA WAN DOU	0.33	5	1
147	G0003273	Hubei, China	MA WAN DOU	0.25	6	1
148	G0003307	Guizhou, China	SHI SHOU KE WAN	0.50	7	3
149	G0003308	Guizhou, China	MAO KOU KE WAN	0.34	4	1
150	G0003309	Guizhou, China	MA ZHAI DA BAI WAN	0.20	4	3
151	G0003315	Guizhou, China	MA JIANG DA BAI WAN	0.26	7	1

152	G0003333	Guizhou, China	MEI MAN CAI WAN DOU	0.47	8	3
153	G0003340	Guizhou, China	XIAO ROU WAN	0.42	8	3
154	G0003341	Guizhou, China	SHI SHOU ROU WAN	0.47	7	3
155	G0003356	Gansu, China	HUA NAI WAN DOU	0.35	6	3
156	G0003468	Greece	K-129	0.50	9	3
157	G0003543	Sichuan, China	II-101	0.59	8	1
158	G0003556	Sichuan, China	2-241	0.21	6	1
159	G0003565	Sichuan, China	921329-1	0.66	9	1
160	G0003566	Sichuan, China	ZI WAN 387	0.56	8	1
161	G0003567	Sichuan, China	2-230	0.32	8	1
162	G0003581	Tibet, China	BAI WAN DOU	0.18	4	1
163	G0003621	Tibet, China	WAN DOU 1 HAO	0.23	5	1
164	G0003645	Tibet, China	MA WAN DOU	0.47	7	1
165	G0003646	Tibet, China	MA WAN DOU	0.48	9	1
166	G0003653	Tibet, China	GONG WAN 1 HAO	0.47	8	1
167	G0003737	Chongqing, China	DA BAI WAN	0.39	5	1
168	G0003744	Sichuan, China		0.35	5	1
169	G0003759	Sichuan, China	BAI WAN	0.11	3	1
170	G0003764	Sichuan, China	DA BAI WAN	0.19	6	1
171	G0003772	Sichuan, China		0.06	3	1
172	G0003776	Sichuan, China		0.13	3	1
173	G0003779	Sichuan, China	YANG WAN	0.34	4	1
174	G0003780	Sichuan, China	YANG MAO WAN	0.26	6	1
175	G0003787	Sichuan, China		0.18	4	1
176	G0003795	Sichuan, China		0.12	3	3
177	G0003803	Sichuan, China	MA WAN	0.18	3	1
178	G0003805	Sichuan, China	MA WAN	0.17	5	1
179	G0003811	Sichuan, China		0.30	6	1
180	G0003853	Shaanxi, China	SHAN WAN DOU 1	0.42	8	3
181	G0003854	Shaanxi, China	SHAN WAN DOU 2	0.52	7	3
182	G0003855	Shaanxi, China	SHAN WAN DOU 3	0.50	7	3
183	G0003858	Shaanxi, China	SHAN WAN DOU 6	0.50	8	3
184	G0003859	Shaanxi, China	SHAN WAN DOU 7	0.44	8	1
185	G0003860	Shaanxi, China	SHAN WAN DOU 8	0.55	9	3
186	G0003863	Shaanxi, China	SHAN WAN DOU 11	0.66	9	1
187	G0003864	Shaanxi, China	SHAN WAN DOU 12	0.62	9	3
188	G0003865	Shaanxi, China	SHAN WAN DOU 13	0.45	9	3
189	G0003973	Romania	P157/88	0.39	5	2
190	G0003997	Chongqing, China		0.14	3	1
191	G0004016	Chongqing, China	BAI WAN DOU	0.14	3	1
192	G0004561	Tibet, China	ZANG WAN 69	0.19	5	1
193	G0004628	Tibet, China	ZANG WAN 136	0.24	7	1
194	G0004629	Tibet, China	ZAO SHU HUI WAN	0.14	3	3
195	G0004699	Tibet, China	ZANG WAN 207	0.04	2	1

196	G0004700	Tibet, China	ZANG WAN 208	0.13	4	1
197	G0004801	Australia		0.25	6	2
198	G0004807	Australia		0.40	7	2
199	G0004814	Australia		0.21	4	2
200	G0004864	Australia		0.20	4	2
201	G0004877	Australia		0.29	6	2
202	G0004883	Australia		0.28	7	2
203	G0004897	Australia		0.04	2	2
204	G0004905	Australia		0.17	3	2
205	G0004961	Australia		0.14	3	2
206	G0005036	Australia		0.14	4	2
207	G0005050	Australia		0.33	5	2
208	G0005054	Australia		0.31	6	2
209	G0005055	Australia		0.29	6	2
210	G0005056	Australia		0.16	5	2
211	G0005057	Australia		0.26	5	2
212	G0005091	Australia		0.42		3
213	G0005101	Australia		0.51	7	3
214	G0005271	Jiangsu, China	WZ-3	0.39	5	2
215	G0005291	Jiangsu, China	WZ-24	0.00		2
216	G0005294	Jiangsu, China	WZ-30	0.39	6	3
217	G0005411	Ningxia, China	LV WAN DOU	0.11	6	1
218	G0005419	Ningxia, China	LAN DOU	0.39	8	1
219	G0005527	Beijing, China	ZHONG WAN 6 HAO	0.12	2	2
220	G0005550	Sichuan, China	MA WAN DOU	0.25	7	1
221	G0005557	Sichuan, China	BAI WAN DOU	0.10	2	1
222	G0005562	Sichuan, China	MA WAN DOU	0.25	6	1
223	G0005563	Sichuan, China	DA MA WAN DOU	0.21	5	1
224	G0005566	Sichuan, China	CAI HUA WAN DOU	0.35	7	3
225	G0005569	Sichuan, China	CAI WAN DOU	0.21	4	1
226	G0005571	Sichuan, China	BAI WAN DOU	0.22	5	1
227	G0005573	Sichuan, China	CAI HUA WAN DOU	0.25	5	1
228	G0005577	Chongqing, China		0.26	4	1
229	G0005578	Chongqing, China		0.35	4	1
230	G0005581	Chongqing, China		0.15	5	1
231	G0005587	Chongqing, China		0.23	5	1
232	G0005588	Chongqing, China		0.18	3	1
233	G0005596	Chongqing, China		0.05	2	2
234	G0005597	Chongqing, China		0.30	6	3
235	G0005598	Chongqing, China		0.25	4	3
236	G0005604	Chongqing, China		0.14	5	3
237	G0005605	Chongqing, China		0.33	5	3
238	G0005607	Chongqing, China		0.41	6	1
239	G0005609	Chongqing, China		0.27	5	1

240	G0005611	Chongqing, China		0.23	5	1
241	G0005613	Chongqing, China		0.22	6	1
242	G0005614	Chongqing, China		0.32	6	1
243	G0005615	Chongqing, China		0.26	5	3
244	G0005619	Chongqing, China		0.25	5	1
245	G0005620	Chongqing, China		0.28	5	1
246	G0005621	Chongqing, China		0.13	3	1
247	G0005622	Chongqing, China		0.25	6	1
248	G0005625	Chongqing, China		0.49	6	1
249	G0005626	Chongqing, China		0.30	8	1
250	G0005794	Chongqing, China		0.52	8	1
251	G0005795	Chongqing, China		0.53	8	1
252	G0005805	Chongqing, China		0.46	9	1
253	G0005827	Chongqing, China		0.10	2	2
254	G0005829	Yunnan, China		0.02	2	3
255	G0005830	Yunnan, China		0.04	2	3
256	G0005832	Yunnan, China		0.33	7	3
257	G0005833	Yunnan, China		0.17	3	3
258	G0005835	Yunnan, China		0.33	5	3
259	G0005844	Yunnan, China		0.19	5	3
260	G0005848	Yunnan, China		0.09	4	1
261	G0005864	Yunnan, China		0.17	4	3
262	G0005866	Yunnan, China		0.46	7	3
263	G0005889	Syria	Nadezhda	0.40	8	3
264	G0005899	Syria	Ramonsky 748-67	0.12	2	2
265	G0005904	Syria	L-60	0.02		2
266	G0005909	Syria	Local	0.24	6	2
267	G0005910	Syria	OUM-2	0.15	4	2
268	G0005911	Syria	Malinovka	0.09	3	2
269	G0005973	Australia	ATC1704	0.44	8	2
270	G0005978	Australia	ATC1694	0.38	6	2
271	G0006623	Canada		0.00		2
272	G0006624	Canada		0.13	3	2
273	G0006625	Canada		0.16	4	2
274	G0006626	Canada		0.22	4	2
275	G0006627	Canada		0.01	1	2
276	G0006628	Canada		0.08		2
277	G0006629	Canada		0.00	0	2
278	G0006630	Canada		0.11	3	2
279	G0006631	Canada		0.00		2
280	G0006632	Canada		0.09	2	2
281	G0006633	Canada		0.00	0	2
282	G0006634	Canada		0.02	1	2
283	G0006635	Canada		0.17	4	2

284	G0006636	Canada		0.34	7	2
285	G0006637	Canada		0.20	4	2
286	G0006638	Canada		0.36	6	2
287	G0006639	Canada		0.12	3	2
288	G0006640	Canada		0.22	4	2
289	G0006641	Canada		0.12	2	2
290	PI 103058		PAI WAN TOU	0.15	3	3
291	PI 109866	Venezuela	ARVEJAS VERDES	0.14	5	2
292	PI 116056			0.24	5	2
293	PI 116844	Pakistan	MATTAR	0.15	5	2
294	PI 116944	Afghanistan	Moshong	0.29	5	1
295	PI 117264	Turkey	No. 215	0.09	3	2
296	PI 117998	Brazil	ERVILHA TORTA FLOR ROXA	0.10	3	2
297	PI 118501	Brazil	ERVILHA BRANCA	0.06	1	2
298	PI 121352	India	No. 1891	0.05	3	2
299	PI 124478	Pakistan	Matar	0.16	4	2
300	PI 125839	Afghanistan	MIZHIK	0.14	4	3
301	PI 125840	Afghanistan	Moshong	0.36	6	1
302	PI 134271	Afghanistan	No. 10	0.15	5	1
303	PI 137118		CHINESE PURPLE	0.09	1	2
304	PI 137119		GRAY'S	0.12	2	2
305	PI 140298	Iran	No. 6618	0.11	3	2
306	PI 142775		ALBERGA	0.17	4	2
307	PI 143485		G 24180	0.20	5	3
308	PI 155109		G 12111	0.12	4	2
309	PI 156647	Ethiopia	G 24184	0.06	4	2
310	PI 156720	Japan	THIRTY-DAYS	0.12	3	2
311	PI 162909	Paraguay	L.P. NO. 4	0.07	2	2
312	PI 163126	India	Matar	0.01	1	3
313	PI 163129	India	Matar	0.16	3	2
314	PI 164182	India	Matar	0.03	2	2
315	PI 164548	India	Matar	0.12	2	2
316	PI 164612	India	Patani	0.09	1	2
317	PI 164779	India	Matar	0.07	2	1
318	PI 164971	Turkey	No. 17	0.38	8	2
319	PI 164972	Turkey	No. 48	0.10	3	2
320	PI 165949	India	Matar	0.13	4	1
321	PI 166084	India	Matar	0.45	7	1
322	PI 166159	Nepal	No. 9705	0.35	6	1
323	PI 169603	Turkey	No. 2187	0.06	3	2
324	PI 169608	Turkey	BEZELYA YESIL	0.11	3	2
325	PI 171810	Turkey	No. 6754	0.25	6	2
326	PI 172339	Netherlands	MANSHOLT PLUK	0.09	2	2

327	PI 174921	Nepal	KALAON	0.19	4	1
328	PI 175231	Nepal	KOLUNG	0.15	3	1
329	PI 179450	Syria	No. 9747	0.15	4	2
330	PI 179451	Syria	No. 9749	0.12	2	2
331	PI 179459	Turkey	No. 10072	0.03	2	2
332	PI 179722	India	No. 10898	0.04	4	2
333	PI 179970	India	Matar	0.00		2
334	PI 180329	India	Watana	0.14	4	3
335	PI 180693	Germany	HOHENHEIMER PINK-FLOWERED	0.06	2	2
336	PI 180696	Germany	MAHNDORFER VIKTORIA	0.10	2	2
337	PI 180699	Germany	RIMPAUS GREEN VIKTORIA	0.01	1	2
338	PI 180702	Germany	STRENGS WEIHENSTEPHANER FELDERBSE	0.04	1	2
339	PI 181799	Lebanon	No. 9901	0.02		2
340	PI 181801	Syria	No. 9922	0.03	2	2
341	PI 181958		Homs No. 334	0.23	3	2
342	PI 184130		No. 309	0.02	2	3
343	PI 184784	Guinea	G 1774	0.00	0	2
344	PI 188698	Nigeria	G 7184	0.19	4	2
345	PI 193578	Ethiopia	No. 8508	0.10	3	3
346	PI 193584	Ethiopia	No. 8696	0.08	2	2
347	PI 193590	Ethiopia	No. 8736	0.17		2
348	PI 195020	Ethiopia	No. 9292	0.06	2	2
349	PI 195404	Guatemala	QUEZALTENANGO	0.01	1	2
350	PI 195631	Ethiopia	No. 9886	0.12	2	2
351	PI 197044	Honduras	No. 3005	0.01	1	2
352	PI 197990	Netherlands	VINCO	0.05	2	2
353	PI 198072	Sweden	BRIOART	0.11	2	2
354	PI 198074	Sweden	GORSDAGSART III	0.04	1	2
355	PI 198735		G 6748	0.26	7	1
356	PI 200755	Guatemala	No. 3498	0.12	2	2
357	PI 201390	Mexico	No. 3153	0.14	2	2
358	PI 203064		G 6820	0.12	2	2
359	PI 203067		G 6823	0.08	3	2
360	PI 203069		G 6825	0.01		2
361	PI 204306	Australia	DUNN	0.03	2	2
362	PI 206006	Sweden	DEBUT 149	0.05	1	2
363	PI 206686			0.00	0	2
364	PI 206838	United States	EVERBEARING	0.00	0	2
365	PI 207508	Afghanistan	No. 12616	0.04	2	1
366	PI 209507	Costa Rica	No. 3843	0.08	2	2
367	PI 210558	China	No. S-653	0.11	3	2

368	PI 210568	Finland	KELINA	0.08	1	2
369	PI 210569	Finland	SIVIKKA	0.11	1	2
370	PI 210571	Finland	FLO	0.00	0	2
371	PI 212031	Iran	No. 4	0.04	1	2
372	PI 212917	India	VATANA MATAR	0.12	3	2
373	PI 220174	Afghanistan	No. 13	0.21	4	1
374	PI 220189	Afghanistan	Moshong	0.26	5	1
375	PI 221697		No. 12	0.03	2	2
376	PI 222071	Afghanistan	Moshong	0.37		1
377	PI 222117	Afghanistan	Moshong	0.23	5	1
378	PI 223527	Afghanistan	LOBIA	0.24	5	2
379	PI 227258	Iran	No. 14918	0.46	7	1
380	PI 236492	United States	LAMPRECHT #368	0.16	3	3
381	PI 240516	India	LUCKNOW BONIYA	0.05	1	2
382	PI 241593	Taiwan, China	G 6571	0.01	1	2
383	PI 242027			0.16	3	2
384	PI 242028	Denmark		0.01	1	2
385	PI 244093	Netherlands	APOLLO	0.03	2	2
386	PI 244121	Netherlands	CRESCENT	0.01	1	2
387	PI 244150	Netherlands	GOLDKONIGIN	0.04	1	2
388	PI 244175	Netherlands	LAGE SMELTPEUL	0.14	3	2
389	PI 244191	Netherlands	MORGENSTER	0.00	0	2
390	PI 248181	Rwanda	Col. No. 23171	0.00	1	2
391	PI 249645		B.R. 178	0.03	2	3
392	PI 250438	Czech Republic	LIBOCHOVICKY RANY	0.13		2
393	PI 250439	Czech Republic	KONSERVOVA KRALOVNA (CSR)	0.00	0	2
394	PI 250440	Czech Republic	KARLOVNA KONSERVY	0.14	3	2
395	PI 250441	Czech Republic	PREBOHATY	0.00	0	2
396	PI 250444	Czech Republic	ZAZRAK Z KELVEDONU	0.00	0	2
397	PI 250446	Czech Republic	LIBOCHOVICKY URODNY	0.05	2	2
398	PI 250448	Czech Republic	LIBOCHOVICKY RANY	0.01		2
399	PI 253968	Afghanistan	Col. No. K1722	0.28	6	1
400	PI 257244	China	TUNG HAUN	0.07	1	2
401	PI 257592	Ethiopia	G 9173	0.08	1	2
402	PI 261623	Spain	GUISANTE PIRABESQUE	0.09	3	2
403	PI 261624	Spain	Col. No. D-45	0.00	0	2
404	PI 261636	Spain	No. D-88	0.07	2	2
405	PI 261671	Netherlands	DEGRACE	0.08	1	2
406	PI 263027	France	MANGETOUT CORNE DE BELEIR	0.07		2
407	PI 263030	France	POIS DU CHEMEN LONG	0.02	1	2
408	PI 263031	France	MANGETOUT CAROUBY	0.26	4	2
409	PI 266070	Sweden	LINE NO. 930	0.07	1	2

410	PI 269543	Pakistan	Col. No. 431	0.05	2	2
411	PI 269761			0.25	6	2
412	PI 269762			0.15	3	2
413	PI 269777	United Kingdom	Aa87	0.08	2	2
414	PI 269778	United Kingdom	Aa88	0.03	1	2
415	PI 269782	United Kingdom	Aa92	0.07	2	2
416	PI 269791	United Kingdom	BROCHETTE	0.11	2	2
417	PI 269802	United Kingdom	Aa112	0.22	4	2
418	PI 269804	United Kingdom	Aa117	0.13	2	2
419	PI 269812	United Kingdom	Aa128	0.00	0	2
420	PI 269818	United Kingdom	Aa134	0.75	9	1
421	PI 269821	United Kingdom	WILLIAM MASSEY, LINE 3	0.00		2
422	PI 271033			0.02	1	3
423	PI 271035	Sweden	HINDUKUSCH	0.01		2
424	PI 271038	Nepal	GRANART	0.00		2
425	PI 271116			0.12	2	2
426	PI 271118			0.29	7	3
427	PI 272148	Finland	NAVALE ARTTURI	0.10	3	2
428	PI 272152	Greece	HIEMALE LANDSORTE	0.35	5	2
429	PI 272171	Germany	ZEYLANICUM, SELECTAS PELUSCHKE	0.13	3	2
430	PI 272175	Germany	ZEYLANICUM	0.13	3	3
431	PI 272194	Germany	CONCOLON	0.11	1	2
432	PI 272204	Germany	HODINGER FUTTERERBSE	0.06	1	2
433	PI 272215	Germany	POPULATION LUCIENHOFER WINTERERBSE	0.09	1	2
434	PI 272216	Bulgaria	WINTER-FUTTERERBSE	0.17	5	2
435	PI 272217	Germany	WURTEMBERGISCHE WI-ERBSE	0.54	9	2
436	PI 272218	Poland	POPULATION	0.49	7	3
437	PI 273209			0.14	5	3
438	PI 273605	Ecuador	G 11058	0.06	2	2
439	PI 274307	Pakistan	809	0.04	2	1
440	PI 274308	Pakistan	815	0.06	2	1
441	PI 274584	Norway	G 11173	0.15	3	2
442	PI 275821	Sweden	G 11250	0.00	1	2
443	PI 275822	Sweden	WITHAM WONDER	0.01	1	2
444	PI 275825	Sweden	G 11254	0.13	2	2
445	PI 275826	Sweden	G 11255	0.16	5	2
446	PI 277852	Ethiopia	No. 8090	0.13	4	2
447	PI 279825	Germany	FOLI NO. 2	0.13	2	2
448	PI 279827	Germany	MANSHOLT'S PLUK	0.09	2	2
449	PI 280252	Ethiopia	No. 8120	0.10	3	2

450	PI 280252	Ethiopia	No. 8120	0.10	3	2
451	PI 280609	Russian Federation	AMPLISSIMO LOCAL	0.38		3
452	PI 280611	Ukraine	AMPLISSIMO VIKTORIA UKRAINSKAYA	0.00	0	2
453	PI 280614	Russian Federation	AMPLISSIMO NEISTOSCIMYJ	0.00		2
454	PI 280616	Russian Federation	AMPLISSIMO ZAZERSKIJ	0.09	2	2
455	PI 280617	Estonia	AMPLISSIMO HAMISEPP	0.16	4	2
456	PI 280619	Russian Federation	AMPLISSIMO TYGEVSKIJ KIRJU	0.23	4	2
457	PI 285710	Poland	KUJAWSKI POZNY	0.22	4	2
458	PI 285717	Poland	NIEZNANICKA CIEMNA	0.22	3	2
459	PI 285718	Poland	POMORSKA	0.27	4	2
460	PI 285719	Poland	PRZEBEDOWSKA OLIWKOWA	0.19	4	2
461	PI 285727	Poland	MAJOWY	0.00	0	2
462	PI 285730	Poland	CUD AMERYKI	0.00	0	2
463	PI 285737	Poland	FOLI	0.00		2
464	PI 285739	Poland	JUNE WONDER	0.11	1	2
465	PI 286430	Nepal	G 12600	0.35	5	3
466	PI 286431	Nepal	Matar	0.11	1	2
467	PI 286607	Thailand	G 12661	0.07	2	2
468	PI 288025	France	QUIKI	0.15	2	2
469	PI 288263	Germany	MARK-ERBSEN	0.00		2
470	PI 306591	Hungary	PJELJUSKA PASSZKAJA	0.22	7	3
471	PI 307666	Costa Rica	VERJA	0.07	1	2
472	PI 308796	India	SYLVIA	0.06	3	2
473	PI 314794	Australia	CPI 12559	0.13	4	2
474	PI 319374	Mexico	Col. No. 22074	0.28	6	2
475	PI 320972	Hungary	IREGI SARGA IP.1	0.07	3	2
476	PI 324695	Hungary	ARAKASS	0.13	3	2
477	PI 324697	Hungary	GRIS D'HIVER	0.24	4	2
478	PI 324702	Hungary	UNRRA/972/	0.48	9	2
479	PI 324703	Hungary	WUNTERBERGISCHE-WINTERERBSE POPULATION	0.62	9	2
480	PI 324706	Romania	No. 833	0.15	4	2
481	PI 331413	Ethiopia	Col. R-44	0.08	3	2
482	PI 331414	Ethiopia	Col. 795-B	0.14	3	2
483	PI 340128	Turkey	1-301	0.29	7	2
484	PI 340130	Turkey	1-305	0.36	7	3
485	PI 341889	Netherlands	ELWY	0.00	0	2

486	PI 343292	United States	G 18305	0.20	6	2
487	PI 343321	United States	G 18451	0.10	1	2
488	PI 343331	United States	G 18461	0.08	2	2
489	PI 343338	United States	G 18662	0.23	3	2
490	PI 343824	Uganda	Col. 6946	0.08	2	2
491	PI 343958	Turkey	BISELIA	0.22	3	2
492	PI 343987			0.18	3	2
493	PI 344003			0.17	3	2
494	PI 344010			0.26	5	3
495	PI 344538			0.23		3
496	PI 347281	India	PLP 10	0.17	5	2
497	PI 347295	India	PLP 26	0.14	4	2
498	PI 347457	India	PLP 264	0.11	2	2
499	PI 347477	India	PLP 503	0.14	2	2
500	PI 347490	India	PLP 82	0.12	2	2
501	PI 347496	India	PLP 105A	0.10	1	2
502	PI 355906	Japan	KOMIDORI	0.20	4	2
503	PI 356973	India	PLP 35	0.18	3	2
504	PI 356974	India	PLP 120	0.09	2	2
505	PI 356984	India	PLP 70	0.05	1	3
506	PI 356986	India	PLP 174	0.12	4	2
507	PI 356991	India	PLP 196	0.16	4	2
508	PI 356992	India	PLP 197A	0.14	3	2
509	PI 357290	Former Serbia and Montenegro	BRZAK	0.00		2
510	PI 358300	Ethiopia	27b	0.22		2
511	PI 358620	Ethiopia	22758	0.07	3	2
512	PI 358633	Ethiopia	22778	0.11	3	2
513	PI 358640	Ethiopia	22791	0.05	2	2
514	PI 365419	Canada	BR 1-49-9	0.20	3	2
515	PI 371796	New Zealand	G 22442	0.22	3	2
516	PI 378157	Malaysia	RED FLOWER NO. 1	0.09	1	2
517	PI 381334	Netherlands	IMPOSANT BROWN	0.21	4	2
518	PI 404225	Russian Federation	PINSKIJ MESTNYJ	0.25	4	2
519	PI 409031	Germany	HOHENHEIMER ROSABLUEHENDE	0.12	3	2
520	PI 413678	Hungary	ZLOTY OLBRZYMI	0.00		2
521	PI 413683	Hungary	LANCET	0.00	0	2
522	PI 413685	Hungary	NZ 51	0.19	4	2
523	PI 413703	Hungary	I.P. 2	0.10	2	2
524	PI 429839	Afghanistan	MUSUS	0.11	3	3
525	PI 429843	Russian Federation	TORSDAG	0.06	3	2

526	PI 429845	Russian Federation	KOMBAJNOVYJ 5	0.10	2	2
527	PI 476409	Latvia	ROTA	0.15	3	2
528	PI 476410	Ukraine	MIRONOVSKIJ 186	0.00	0	2
529	PI 476413	Russian Federation	UL'IANOVSKIJ-72	0.06	2	2
530	PI 477371	Denmark	ROSAKRONE	0.16		2
531	PI 486131	Ecuador	E8454-A-F	0.09	1	2
532	PI 494077	Chile	G 27915	0.17		2
533	PI 499982	China	G 28097	0.29		1
534	PI 505059			0.11		3
535	PI 505080			0.14	3	2
536	PI 505108			0.65	8	3
537	PI 505127			0.26	5	2
538	PI 505144			0.24	4	2
539	PI 560071			0.00		3
540	PI 639964			0.11	2	2
541	PI 639967			0.33	6	1
542	PI 639977			0.50	8	2
543	PI 639980	Bulgaria		0.41	9	2
544	PI 639981			0.43	7	2
545	PI116844	Pakistan	MATTAR	0.12	2	2
546	PI116944	Afghanistan	Moshong	0.14	6	1
547	PI117264	Turkey	No. 215	0.05	2	2
548	PI117998	Brazil	ERVILHA TORTA FLOR ROXA	0.13	4	2
549	PI118501	Brazil	ERVILHA BRANCA	0.16	3	2
550	PI120623	Turkey	No. 545	0.16	3	2
551	PI121976	India	SHANDIL	0.10	2	1
552	PI125672	Austria	G 1696	0.10	4	2
553	PI126341		G 24178	0.31	7	1
554	PI138945	Iran	KUDI	0.11	5	3
555	PI140295	Iran	CN 43018	0.06	4	2
556	PI140296	Iran	No. 6182	0.36	6	3
557	PI163130	India	Matar	0.17	3	2
558	PI163134	India	Matar	0.29	6	2
559	PI164417	India	No. 8710	0.24	4	2
560	PI164614	India	Patani	0.25	6	2
561	PI164690	India	No. 9116	0.10	4	2
562	PI167204	Turkey	BEZELYE	0.27	4	2
563	PI169609	Turkey	No. 3392	0.35	4	2
564	PI169610	Turkey	No. 3765	0.16	4	2
565	PI172341	Netherlands	UNICA	0.14	3	2
566	PI173057	Turkey	No. 8103	0.22	5	2

567	PI174320	Turkey	SULTANI	0.24	4	2
568	PI174321	Turkey	No. 8459	0.10	3	2
569	PI174322	Turkey	No. 8773	0.18	5	2
570	PI174920	India	KOSHI	0.24	3	1
571	PI174922	India	No. 9625-A	0.14	3	1
572	PI174925	India	Matar	0.03	2	2
573	PI175233	India	Matar	0.26	4	2
574	PI177053	Turkey	No. 5366	0.15	5	2
575	PI177055	Turkey	No. 5899	0.11	4	2
576	PI177056	Turkey	No. 9319	0.19	4	2
577	PI179019	Turkey	No. 9618	0.21	6	2
578	PI179969	India	LAMBU KRA	0.36	6	1
579	PI180695	Germany	LUCIENHOFER	0.35	6	2
580	PI180868	Turkey	No. 9593	0.37	8	2
581	PI184131		No. 310	0.19	4	2
582	PI194349	Ethiopia	No. 9105	0.22	4	2
583	PI215766	Peru	ALRECYOS	0.07	3	2
584	PI221514	Afghanistan	Moshung-Khord	0.25	4	1
585	PI222069	Afghanistan	Moshong	0.11	2	1
586	PI222117	Afghanistan	Moshong	0.25	5	1
587	PI244095	Netherlands	AUREOOL	0.09	3	2
588	PI244189	Netherlands	MOERHEIMS REUZEN	0.02		2
589	PI261635	Spain	No. D-87	0.20	4	2
590	PI267860	Netherlands	RONDO C.B.	0.04	1	1
591	PI271034	Sweden	ASIATICUM	0.29	4	1
592	PI271036	Sweden	TRANSCAUCASICUM	0.29	4	1
593	PI271121	Germany	G 3586	0.26	5	2
594	PI272146	Germany	HIBERNICUM	0.36	5	2
595	PI272149	Germany	NAVALE	0.35	6	2
596	PI272150	Germany	ANKOBERENSE	0.30	5	2
597	PI272157	Greece	HIEMALE	0.29	4	2
598	PI272166	Greece	GRISEO COLORATUM	0.28	4	2
599	PI272176	Germany	PUNCTATUM	0.18	5	2
600	PI272178	Germany	SUBHARRARICUM	0.14	5	2
601	PI272193	Germany	NIGRO-VIOLACEUM	0.13	4	2
602	PI272202	Germany	ALVEOLARE	0.14	3	2
603	PI272217	Germany	WURTEMBERGISCHE WI-ERBSE	0.63	7	2
604	PI273675	Ethiopia	1867	0.07	2	2
605	PI311112	Guatemala	Col. No. 21386	0.08	2	2
606	PI314797	Australia	CPI 15248	0.28	5	2
607	PI318434	United Kingdom	SUTTON'S CHIEFTAIN	0.24		2
608	PI330383	Netherlands	Flavanda	0.02	2	2
609	PI330384	Netherlands	Porta	0.20	4	2

610	PI339887	Turkey	Karagoz	0.23	6	2
611	PI343276	United States	G 18287	0.33	5	2
612	PI343325	United States	G 18455	0.26	5	2
613	PI347300	India	PLP 32	0.15	4	2
614	PI347303	India	PLP 43	0.10	3	2
615	PI347304	India	PLP 45A	0.09	4	2
616	PI347316	India	PLP 68	0.19	5	2
617	PI347319	India	PLP 73	0.13	5	2
618	PI347324	India	PLP 90	0.07	4	2
619	PI347332	India	PLP 104	0.03	2	2
620	PI347339	India	PLP 127	0.11	3	2
621	PI347340	India	PLP 130	0.08	3	2
622	PI347341	India	PLP 137	0.08	3	2
623	PI347342	India	PLP 154	0.12	4	2
624	PI347349	India	PLP 188	0.21	6	2
625	PI347352	India	PLP 212	0.10	3	2
626	PI347360	India	PLP 227	0.03	2	2
627	PI347361	India	PLP 234	0.04	2	2
628	PI347365	India	PLP 265	0.06	2	2
629	PI347373	India	PLP 301	0.09	2	2
630	PI347390	India	PLP 333	0.23	5	2
631	PI347407	India	PLP 372	0.13	3	2
632	PI347413	India	PLP 390	0.02	1	2
633	PI347446	India	PLP 86	0.09	4	2
634	PI347480	India	PLP 37	0.18	4	2
635	PI347499	India	PLP 124	0.03	2	2
636	PI347508	India	PLP 146	0.13	3	2
637	PI356835	India	PLP 50	0.10	3	2
638	PI356839	India	PLP 75	0.14	3	2
639	PI356840	India	PLP 76	0.18	6	2
640	PI356842	India	PLP 84	0.04	3	2
641	PI356855	India	PLP 171	0.22	4	2
642	PI356856	India	PLP 172	0.08	3	2
643	PI356861	India	PLP 180	0.21	4	2
644	PI356863	India	PLP 185	0.05	3	2
645	PI356865	India	PLP 189	0.25	5	2
646	PI356866	India	PLP 190	0.19	4	2
647	PI356879	India	PLP 211	0.13	4	2
648	PI356881	India	PLP 224	0.05	3	2
649	PI356884	India	PLP 228	0.18	3	2
650	PI356885	India	PLP 229	0.22	4	2
651	PI356889	India	PLP 236	0.21	4	2
652	PI356890	India	PLP 238	0.31	4	2
653	PI356893	India	PLP 250	0.13	3	2

654	PI356896	India	PLP 257	0.10	3	2
655	PI356898	India	PLP 263	0.15	4	2
656	PI356899	India	PLP 266A	0.11	3	2
657	PI356900	India	PLP 270	0.15	3	2
658	PI356922	India	PLP 376	0.17	4	2
659	PI356927	India	PLP 394	0.18	3	2
660	PI356928	India	PLP 395	0.15	4	2
661	PI356929	India	PLP 397	0.17	4	2
662	PI356934	India	PLP 407	0.11	2	2
663	PI356941	India	PLP 431	0.18	4	2
664	PI356943	India	PLP 434	0.18	4	2
665	PI356945	India	PLP 445	0.20	3	2
666	PI356954	India	PLP 490	0.16	3	2
667	PI356976	India	PLP 129	0.11	1	2
668	PI356978	India	PLP 140	0.10		2
669	PI358666	Ethiopia	22829	0.18	3	2
670	PI358667	Ethiopia	22830	0.26	4	2
671	PI358672	Ethiopia	22836	0.25	3	2
672	Q084			0.40	6	2
